# Neurotoxicity, Behavior, and Lethal Effects of Cadmium, Microplastics, and Their Mixtures on *Pomatoschistus microps* Juveniles from Two Wild Populations Exposed under Laboratory Conditions―Implications to Environmental and Human Risk Assessment

**DOI:** 10.3390/ijerph16162857

**Published:** 2019-08-10

**Authors:** Tiago Miranda, Luis R. Vieira, Lúcia Guilhermino

**Affiliations:** 1 ICBAS—Institute of Biomedical Sciences of the University of Porto, Department of Populations Study, Laboratory of Ecotoxicology (ECOTOX), 4050-313 Porto, Portugal; 2 CIIMAR—Interdisciplinary Centre of Marine and Environmental Research of the University of Porto, Research Team of Ecotoxicology, Stress Ecology and Environmental Health (ECOTOX), 4450-208 Matosinhos, Portugal

**Keywords:** estuarine fish, cadmium, microplastics, behavior, neurotoxicity, inter-population variability, median lethal concentrations, environmental and human risk assessment

## Abstract

Microplastics (MPs) were found to modulate the toxicity of other pollutants but the knowledge on the topic is still limited. The goals of this study were to investigate the short-term toxicity of cadmium (Cd) to wild *Pomatochistus microps* juveniles, the potential modulation of acute Cd toxicity by 1–5 µm polyethylene MPs in this species, and possible differences of sensitivity to Cd and MPs-Cd mixtures between juveniles from two distinct wild populations. Juveniles were collected in the estuaries of Minho (M-est) and Lima (L-est) Rivers (NW Portugal). One 96 h bioassay with M-est juveniles and another one with L-est juveniles were carried out in laboratory conditions. Each bioassay had 12 treatments: control, 5 Cd concentrations, 1 MPs concentration, and 5 MPs-Cd mixtures. No significant differences in Cd-induced mortality between juveniles from distinct estuaries or between juveniles exposed to Cd alone and those exposed to MPs-Cd mixtures were found. The total 96h LC_10_ and LC_50_ of Cd alone were 2 mg/L (95% CI: 0–4 mg/L) and 8 mg/L (95% CI: 2–17 mg/L), respectively. Cd alone significantly decreased the post-exposure predatory performance (PEPP) of M-est (≥6 mg/L) and L-est juveniles (≥3 mg/L), and acetylcholinesterase (AChE) activity of M-est juveniles (13 mg/L). MPs alone (0.14 mg/L) significantly reduced the PEPP and AChE activity of L-est juveniles but not of M-est juveniles. MPs-Cd mixtures (3–13 mg/L of Cd + 0.14 mg/L of MPs) significantly inhibited the PEPP of juveniles from both estuaries and AChE of L-est estuary juveniles but not of M-est juveniles. Evidences of toxicological interactions, namely antagonism, between MPs and Cd were found. Overall, the results indicate that MPs modulated the sub-lethal toxic effects of Cd in wild *P. microps* juveniles, especially neurotoxicity. Moreover, the environmental conditions of the natural habitats to which juveniles were exposed during pre-developmental phases influence the sub-lethal toxicity of Cd, MPs, and their mixtures. The implications to environmental and human risk assessment are discussed and further research is needed.

## 1. Introduction

Cadmium (Cd) and microplastics (plastic debris <5 mm, hereafter indicated as MPs) are pollutants of high concern regarding ecosystem, animal, and human health that are globally dispersed in marine, freshwater, and terrestrial ecosystems [1,2,3,4,5]. Both pollutants can be transported for long distances mainly through long range air circulation, marine, and freshwater currents and contaminated biota. In general, animals and humans may be exposed to both substances through contaminated food, air, water, sediments, and soil [1,4,5]. Therefore, it is relevant to investigate the potential toxic effects induced by the simultaneous exposure to Cd and MPs.

Cd is a natural element that may be present at high concentrations in certain areas due to natural processes and anthropogenic activities [6,7] and is considered a priority pollutant [8,9]. It is classified as a human carcinogen, has long biological half-life, is bioaccumulated, and causes neurotoxicity and several other toxic effects [1,7,10,11]. Concentrations of Cd in natural waters, suspended particulate matter, and sediments up to the low ppm range are widely documented [12,13,14] but higher concentrations in sediments were also reported [6].

In fish, Cd can cause neurotoxicity [15], oxidative stress [16], olfactory dysfunction [17], immunity disruption [18], genotoxicity [19], behavioral alterations [20], reproductive impairment, mortality, among other toxic effects [21,22,23]. Fish accumulate Cd [12,24] increasing the risk of exposure and toxic effects to their predators and humans consuming contaminated specimens.

Concentrations of MPs up to the low ppm range in the water and sediments of aquatic ecosystems were found [25,26,27]. The MPs present in aquatic ecosystems are diverse [25,28]. Such MPs generally contain a wide range of other chemical substances, including additives added to MPs during plastic production, chemicals adsorbed to MPs during their use in other industrial processes, and environmental contaminants adsorbed to MPs during their permanence in environmental compartments [29,30]. The MPs present in the environment may also contain microorganisms, including human and animal pathogens [4,31].

Fish, including species widely consumed as food by humans, ingest and uptake MPs [32,33,34] in other ways and accumulate them in internal tissues [35,36,37]. After entering into the fish body, MPs and/or the chemicals that they contain are able to cause a wide range of toxic effects, including neurotoxicity [38,39], oxidative stress and damage [40,41,42], adverse intestinal alterations [43,44], changes in nutritional composition and energy reserves [45], behavior alterations [46,47,48], and endocrine disruption [49], among several others [50,51]. Therefore, aquatic species consumed as food by humans may be contaminated by MPs and other chemicals and microorganisms associated to MPs, and they may have a decreased health and nutritional value. Thus, in addition of being a considerable environmental problem, the contamination of aquatic ecosystems by MPs may also be a risk to public health.

Estuaries have a very high conservational and economic value at global scale, and provide most important ecosystem services [52]. They receive environmental contaminants inputs from both marine and continental origins, including MPs [53,54]. Several of these environmental contaminants may sink and concentrate in particular sites of estuaries. Moreover, in several regions, human and industrial settlements are located in the vicinity of estuaries and other coastal ecosystems, contributing to direct inputs of several environmental contaminants. Thus, estuarine biota is often at increased risk of simultaneous exposure to different toxic chemicals such as Cd and MPs.

In the environment, MPs may adsorb and accumulate Cd [55,56]. Therefore, MPs may be an additional route of exposure of the biota and humans to Cd. Additionally, the simultaneous exposure to MPs and Cd may result in toxicological interactions, increased toxicity, and higher Cd bioaccumulation, as found recently in a study with the zebrafish (*Danio rerio*) chronically exposed to Cd and polystyrene MPs [57]. However, reduced toxic effects caused by polystyrene MPs-Cd mixtures and decreased Cd accumulation in fish (*Symphysodon aequifasciatus*) after chronic exposure were also reported [58]. Therefore, the toxic effects of Cd and MPs-Cd mixtures in fish deserve further investigation.

The common goby *Pomatoschistus microps* (Krøyer, 1838) is a common and abundant species in a high number of European coastal ecosystems [59], where it is an important prey for higher level predators, including species consumed by humans [60]. *P. microps* is a good bioindicator [12,61] and a suitable test organism [62,63]. Because in shallow water systems, juveniles feed on both zooplankton and benthic preys, they may be simultaneously exposed to chemical contaminants present in the water and sediments [46,60]. *P. microps* ingests MPs of different sizes and toxic effects induced by exposure to MPs have been found in this species, such as mortality (decrease of the predatory performance and neurotoxicity [38,46,64]. Moreover, *P. microps* accumulates Cd with concentrations reported in fish from polluted estuarine sites in the low ppm range, such as a mean of 4.38 µg/g dry weight (dw) in muscle tissue [12].

The goals of this study were to investigate the short-term toxicity of Cd to wild *P. microps* juveniles, the potential modulation of acute Cd toxicity by polyethylene MPs in this species, and possible differences of sensitivity to Cd and MPs-Cd mixtures between juveniles from two distinct wild populations.

## 2. Materials and Methods

### 2.1. Chemicals

Cadmium chloride (pentahydrate, analytic grade, Merck, Germany) was used as Cd source. Cd was selected as a test substance because it is a ubiquitous pollutant commonly found in estuaries sometimes at increased concentrations [12], is very toxic [1], and its chronic toxicity and bioaccumulation in fish were found to be modulated by polystyrene MPs with some apparent contradictory results [57,58].

MPs were polyethylene microspheres (1–5 µm diameter) from Cospheric – Innovations in Microtechnology, USA. According the indications provided by the manufacturer, their properties were: red opaque color; 1-5 µm of diameter; 1.2 g/cc density; and they were fluorescent (365, 460, and 470 nm excitation and 588 emission wavelengths). These particles were selected as MPs model mainly because polyethylene is one of the types of plastics most produced and found in marine organisms and in the environment [25], their fluorescence allows easy quantification of exposure concentrations, their toxicity to *P. microps* was previously investigated [60,63] providing some basic knowledge, and more knowledge on the toxicity of MPs in the low micro-size range and on their toxicological interactions with other common pollutants is needed [4].

The salt used to prepare the artificial water for fish acclimatization and testing was the Ocean Fish salt from PRODAC, Italy. The Bradford reagent was from Bio-Rad (Germany), and all the other chemicals were of the highest purity available and purchased from Sigma-Aldrich (Germany) or Merck (Germany).

### 2.2. Ethical Issues

The collection of fish in wild populations and the experimentation had authorization from the Portuguese Competent Authorities, including the Portuguese National “Instituto da Conservação da Natureza e das Florestas” (license numbers: 725/2016/CAPT and 617/2017/CAPT) and the Portuguese National “Direção Geral de Alimentação e Veterinária”—DGAV (DGAV reference: 0421/000/000/2017; 2017-05-31, 014227), through the project PLASTICGLOBAL (Portuguese “Fundação para a Ciência e a Tecnologia”: PTDC/MAR-PRO/1851/2014; European Regional Development Fund: COMPETE 2020 programme POCI-01-0145-FEDER-016885, and LISBOA 2020 programme LISBOA-01-0145-FEDER-016885). The experiment followed the ethical principles and regulation regarding animal experimentation (European Union and Portugal), except regarding chemical anesthetics that were not used to avoid potential interference with the tested substances and reagents used for biomarkers determination [46]. Cold-induced anesthesia was used instead. L. Guilhermino and L. R. Vieira have accreditation to conduct animal experimentation (FELASA C equivalent, DGAV, Portugal).

### 2.3. Collection of Specimens and Samples

Sampling of *P. microps* juveniles was performed in the estuaries of Minho River (M-est) and Lima River (L-est). These estuaries were selected mainly because their *P. microps* populations were previously investigated in biomonitoring studies [61,65] and toxicity bioassays and found to have several differences, including in their relative sensitivity to some environmental contaminants such as MPs [46,64]. Moreover, M-est and L-est are relatively close located, have comparable conditions regarding several abiotic conditions but they also have important differences in others, including in the water, sediments and biota concentrations of several environmental contaminants [65,66]. In general, and considering the lower area of the estuaries, the L-est is more contaminated than the M-est [65].

The sampling sites were ~41°53’31” N, 8°49’28” W in the M-est and ~41°41’11.41” N, 8°49’20.42” W in the L-est, corresponding approximately to sites R2 and S1, respectively, of a previous monitoring programme carried out with *P. microps* populations from M-est and L-est [65], where a description of the sampling sites is provided. The annual mean (± standard error of the mean—SEM) of cadmium concentrations in sediments determined in that monitoring programme were 0.01 ± 0.00 µg/g dry weight (dw) in R2 and 0.06 ± 0.02 µg/g dw in S1 and were significantly (*p* < 0.05) different [65]. The corresponding concentrations (whole body) in *P. microsps* juveniles (2–4 cm total body length) were also significantly different but followed an opposite trend: 0.03 ± 0.01 in M-est fish and 0.01 ± 0.00 µg/g dw in L-est fish [65].

For the present study, *P. microps* early juveniles (0+ age group, 108 per estuary) with about ~ 2–2.5 cm of total length were collected at low tide with a hand operated net. *P. microps* specimens with other sizes and other organisms present in the net were immediately and carefully removed and put back into the water. Fish were transported to the laboratory as soon as possible in thermally isolated boxes with water from the respective collection site.

### 2.4. Acclimatization of Fish to Laboratory Conditions

The juveniles were acclimatized to laboratory conditions for 14 days. First, they were gradually (4 days) acclimatized to artificial salt water (commercial marine salt from PRODAC, Italy, dissolved in distilled water and salinity adjusted to 18 g/L, hereafter indicated as test medium), in 60 L glass aquaria with continuous air supply as indicated in previous studies [46,60]. Test medium temperature ranged from 20 °C to 24 °C, an ecological relevant range of temperature variation in the M-est and L-est and several other ecosystems of temperate, sub-tropical, and tropical regions [66,67]. The photoperiod was 16 h light: 8 h dark (~late Spring and Summer photoperiod in several South European areas). Juveniles were further acclimatized to these conditions for 10 additional days, with test medium renewed 3 times a week. During the acclimatization, juveniles were feed *ad libitum* with commercial fish food (AQUAPEX, Portugal), and the quality of water as monitored. Test medium temperature, pH, salinity, and dissolved oxygen were measured at the time of medium renewal (HACH 40d field case probe, USA; HANNA HI digital refractometer, USA). The concentrations of Cd and MPs in juveniles after capture, after acclimatization, and at the end of the bioassays were not determined because the juveniles were very small and therefore such determinations will require a considerable number of additional juveniles for such analyses only.

### 2.5. Exposure Conditions of the Bioassays

The feeding of juveniles was stopped 24 h before the starting of the bioassays. Two 96 h bioassays were carried out in static conditions (i.e., no test medium renewal): one with M-est juveniles and the other with L-est juveniles. They were carried generally according to OECD guidelines for acute testing with juvenile fish [68] with some modifications as further described, under environmental conditions similar to those described for the acclimatization period including the same test medium. No food was provided during the exposure period. Due to the high sensitivity of early *P. microps* juveniles to manipulation [46], specimens were measured and weighted at the end of the bioassays.

In each bioassay, juveniles were exposed individually for 96 h in glass beakers with 500 mL of test medium and continuous additional air supply [63]. Nine juveniles per treatment were used. The treatments were: control (test medium only); MPs (0.18 mg/L), 3 mg/L of Cd; 6 mg/L of Cd; 13 mg/L of Cd; 25 mg/L of Cd; 50 mg/L of Cd; 5 microplastic-cadmium (MPs-Cd) mixtures containing each one the Cd concentrations previously indicated and 0.18 mg/L of MPs.

The concentrations of MPs and Cd tested were selected based on previous studies [38,46,60,69] and on the published literature. They were selected with the main goal of investigating the potential influence of MPs on Cd acute toxicity to juveniles inhabiting different estuaries. The concentrations of MPs in environmental waters reported in the literature and generally referring to particles larger than those used here are diverse [25,28] but high concentrations of MPs such as 5.51 ± 9.09 mg/L in polluted waters were already reported [26]. Moreover, organisms can accumulate MPs [70]. Thus, even considering differences of particles’ size, the concentration of MPs tested in the present study can be considered ecologically relevant.

Regarding Cd, the tested concentrations are higher than those generally reported for natural waters. However, the water column suspended particular matter, sediments, and fish may have considerable concentrations of Cd. For example, mean Cd concentrations of 6.44 µg/g (dw) in the water column suspended particular matter, of 2.63 µg/g (dw) in bottom sediments, and up to 6.92 µg/g in the biota were found in the Seixal bay (Tagus River estuary, Portugal) [12]; mean Cd concentrations up to 19 ± 2 mg/Kg (dw) were found in sediments of the Southern Atlantic coast of Spain [71]; and mean Cd concentrations between 0.9 and 56 mg/Kg (dw) were determined in the liver of the sardinelle (*Sardinella aurita*) from the Senegalese coast [72].

Treatments containing Cd alone were prepared by serial dilution in test medium of a stock solution containing 50 g/L of Cd in ultra-pure water. A stock solution of MPs (supplied as powder) with a concentration of 18 mg/L was prepared in test medium. The treatment of each bioassay containing MPs alone was prepared by dilution of the appropriate volume the stock solution in test medium to reach the nominal concentration of 0.18 mg/L. Mixture treatments were prepared by serial dilution in test medium of the appropriate volumes of the stock solutions of Cd and MPs. No dispersants were used to prepare the treatments containing MPs to have conditions more similar to those occurring in estuarine ecosystems.

Effect criteria were mortality, and the following biomarkers: post-exposure predatory performance (hereafter indicated as predatory performance), acetylcholinesterase (AChE), and glutathione *S*-transferases (GST) activity, and lipid peroxidation (LPO) levels. Predatory performance, AChE activity, and GST activity were selected because they are crucial for fish survival and fitness [46]. Moreover, Cd can change GST activity in fish [19]. Furthermore, in fish, Cd and some MPs were found to inhibit AChE activity [38,73] and cause oxidative stress [16,40,41], 2018b,c) and behavior disruption [20,46,47].

During the exposure period, juveniles were observed at least twice a day including in relation to signs of stress and suffering. Dead juveniles were removed as soon as they were noticed. Test medium temperature, pH, salinity, and dissolved oxygen were determined (as previously indicated in Section 2.3) at the beginning of the bioassays and at 24 h intervals up to 96 h.

Samples of test medium from treatments containing MPs were collected at beginning (0 h) and at the end (96 h) of the exposure period to determine their actual concentrations of MPs. The actual concentrations of MPs tested were determined by spectrofluorimetry [46] using the linear regression model indicated in a previous study of our team where the same lot of microplastics was used [63]:

MPs concentration (mg/L) = −0.124 + 0.013 × fluorescence (F units)

The detailed procedure used to model determination is described in [63], supplementary material. Briefly, 3 independent stock solutions of MPs (1.5 mg/L) in test medium were prepared. Each one was serial diluted (1:2 volume/volume) to obtain 3 series of solutions with MPs concentrations ranging from 1.5 to 0.012 mg/L. The fluorescence of the solutions was read (470 nm excitation and 588 emission wavelengths according to MPs manufacturer in a JASCO, FP-6200 spectrophotometer, USA) immediately after preparation, plotted against the corresponding nominal MPs concentrations and a linear regression model was adjusted to the data. At the beginning and at the end of the bioassays, the fluorescence of test medium of treatments containing MPs was read and the actual MPs concentrations in test medium were calculated from the model.

The decrease of MPs concentrations over time per treatment, hereafter indicated as MPs decay, was calculated [46] as:

MPs decay (%) = 100 − (mean of MPs actual concentration at the end of the bioassay × 100 / mean of MPs actual concentration at the beginning of the bioassay)

Because considerable MPs decay (28–35%) occurred during the bioassays, and no significant differences (*p* > 0.05) between bioassays with juveniles from distinct estuaries or among treatments were found (Table S1), the total MPs midpoint estimated exposure concentration (EEC) was calculated [52] as:

EEC = (total mean of MPs actual concentrations in fresh medium + total mean of MPs actual concentrations in old medium)/2

The value obtained was 0.14 mg/L (Table S1). It was taken as the estimated exposure concentration of MPs during the bioassays, and used to express the biological results.

### 2.6. Determination of Biomarkers

After 96 h of exposure, the predatory performance of each juvenile was determined as indicated in [46] but using 30 nauplii of *Artemia franciscana* (48 h after hatching) as prey for each juvenile. The test is based on the principle that intoxicated organisms have a decreased predatory performance in relation to no-intoxicated ones. Briefly, each juvenile was transferred into a post-exposure predatory-prey chamber containing clean medium. After 5 min, 30 *Artemia* nauplli were introduced into test medium and the number of nauplii ingested by the juvenile was recorded for 3 min. After, the juvenile was removed from the chamber and the number of naupllii remaining in the chamber was counted to verify the number of nauplii ingested by the juvenile. Then, the juveniles used for the predatory performance were measured, weighted, and left resting for 2 h [46]. The resting period allowed juveniles to recover from the post-exposure predatory assay and its duration was selected based on previous studies with *P. microps* juveniles [46,60,63]. After, the same juveniles were sacrificed by decapitation under cold-induced anesthesia. From each juvenile, the head and the remanding body were isolated on ice and kept separately at −80 °C until further analyses. Juveniles were very small and therefore all their body was used for determination of biomarkers. Thus, the potential presence of MPs inside the body of juveniles was not analyzed for ethical reasons as it would require the use of additional fish.

In the day of biomarker analyses, samples were prepared and biomarkers were determined as indicated in [46]. Briefly, the supernatant of head homogenates was used for AChE activity determinations; in this species and in the conditions used the supernatant contains mainly AChE [74]. The remaining body was used for GST activity and LPO determinations. All the determinations were made at 25 °C after protein quantification [75,76] and standardization [46]. The analyses were done in a Biotek, powerwave 340 spectrophotomer (USA), at 25 °C. AChE activity was determined according to the Elman’s technique [77] with some adaptations [76], at 412 nm, using acetylthiocholine as substrate. GST activity was determined following [78] with some adaptations [79], at 340 nm and using 1-chloro-2,4-dinitribenzene as substrate. LPO levels were assessed through quantification of thiobarbituric reactive substances (TBARS) at 535 nm according to Reference [80] with changes [81,82]. At the end of the enzymatic and LPO determinations, the protein content of the samples was quantified [75,76] and used to express the enzymatic activities and LPO levels. Enzymatic activities were expressed in nanomoles of substrate hydrolyzed per minute per mg of protein (nmol/min/mg protein), and LPO levels in nanomoles of TBARS per mg of protein (nmol TBARS/mg protein).

### 2.7. Statistical Analyses

Data were expressed as the mean ± SD or mean ± SEM. Data sets (abiotic and biotic) for Analysis of Variance (ANOVA) were checked for departures of normal distribution (Kolmogorov–Smirnov’s test) and homogeneity of variances (Levene’s test) and transformed when necessary [83]. Data expressed in percentages were arcsine transformed previously to ANOVA [83]. MPs concentrations in different treatments containing these particles were analyzed through a two-way ANOVA (2-ANOVA) with interaction, fixed factors: estuary and treatments (Cd concentrations).

The arcsine transformed percentages of mortality obtained in the two bioassays were first analyzed by two-way between-groups Analysis of Covariance (ANCOVA) after checking for the assumptions of normality, linearity, homogeneity of variances, and homogeneity of regression slopes. The independent variables were the estuary where the juveniles were collected (M-est; L-est) and the presence of MPs in treatments (yes; no), the dependent variable was the arcsine transformed percentages of mortality and the covariate was Cd concentration. To calculate the lethal concentrations of Cd to 10% (LC_10_), 20% (LC_20_) and 50% (LC_50_) of M-est and L-est juveniles after 96 h of exposure to Cd alone and in the presence of MPs, two toxicity curves were obtained for each bioassay: one by plotting the probit transformed percentages of mortality in treatments containing Cd alone versus the log transformed Cd concentrations; the other, by plotting the probit transformed percentages of mortality in treatments containing MPs-Cd mixtures against the log transformed Cd concentrations. Moreover, as no significant differences in Cd induced mortality between M-est and L-est juveniles were found by ANCOVA (as indicated in Section 3.1), two additional curves were made with juveniles of both estuaries: one with the probit transformed percentages of mortality induced by Cd alone versus the log transformed Cd concentrations; the other with the probit transformed percentages of mortality induced by MPs-Cd mixtures versus the log transformed Cd concentrations. One log-probit model [84] was adjusted to each toxicity curve. The 96 h LC_10_, LC_20_ and LC_50_ were calculated from these models, as well as the corresponding 95% confidence interval (95% CI).

For each biomarker, the values determined in M-est and L-est control groups were compared by the Student’s *t* test with equal variances assumed or not assumed as appropriate to each case. Each total biomarker data set (i.e., M-est and L-est juveniles and all treatments) was analyzed through a three-way ANOVA (3-ANOVA) with interactions (fixed factors: estuary from where fish came from, Cd concentrations, and MPs presence/absence). When significant differences between estuaries or significant interactions were found, data were further analyzed per estuary separately through a 2-ANOVA with interaction (fixed factors: Cd concentrations and MPs presence/absence), followed by the Tukey’s multi-comparison test when significant differences among Cd concentrations were found. Predatory performance and AChE activity data analysis of juveniles from each estuary was complemented through the comparison of all individual treatments per biomarker using the Tukey’s multi-comparison test, allowing the determination of the no observed effect concentrations (NOEC) and the lowest observed concentrations (LOEC) that may be of interest for risk assessment.

In all the statistical analyses, the significance level was 0.05. The SPSS statistic package (version 25, IBM, Armonk, NY, USA) was used.

## 3. Results and Discussion

In the test medium of each beaker, the pH variation was lower than 1.0 pH units, the dissolved oxygen was above 8 mg/L. No mortality was recorded in the control groups. These results are in accordance with OECD guidelines for acute testing with juvenile fish [68] regarding these parameters, and indicate that the abiotic conditions during the exposure period were adequate to *P. microps* juveniles.

The total mean (±SD) of length of the M-est juveniles that survived until the end of the bioassay and were used for biomarkers (*N* = 50) was 2.2 ± 0.2 cm, whereas the total mean (±SD) of their weight was 0.11 ± 0.04 g. The total mean (±SD) of length of the L-est juveniles that survived until the end of the bioassay and were used for biomarkers (*N* = 46) was 2.2 ± 0.3 cm, whereas the total mean (± SD) of their weight was 0.12 ± 0.05 g.

The mean (±SD, *N* = 9) of the biological parameters recorded in the control group of the bioassay with M-est juveniles were: 2.2 ± 0.2 cm length; 0.10 ± 0.03 g weight; 89 ± 8% predatory performance; 90 ± 14 nmol/min/mg protein AChE activity, 26 ± 11 nmol/min/mg protein GST activity; and 0.5 ± 0.1 nmol TBARS/mg protein. The corresponding means (±SD, *N* = 9) recorded in the control group of the bioassay with L-est juveniles were: 2.1 ± 0.3 cm length; 0.09 ± 0.04 g weight; 84 ± 14% predatory performance; 78 ± 3 nmol/min/mg protein AChE activity, 22 ± 3 nmol/min/mg protein GST activity; and 0.6 ± 0.3 nmol TBARS/mg protein.

No significant differences of length (*t*_16_ = 0.603, *p* = 0.555), weight (*t*_16_ = 0.469, *p* = 0.645), predatory performance (*t*_12.476_ = 0.773, *p* = 0.454), GST activity (*t*_9.627_ = 1.233, *p* = 0.247), or LPO levels (*t*_9.750_ = −1.080, *p* = 0.306) between juveniles from distinct estuaries were found. Thus, M-est and L-est juveniles were in a comparable developmental stage, and had comparable predatory performance, GST activity, and LPO basal levels.

Significant differences in AChE activity (*t*_8.923_ = 2.686, *p* = 0.025) between the two groups of juveniles were found, with M-est juveniles having significantly higher enzymatic activity than those of the L-est. These results indicate exposure of the L-est population to anticholinesterase agents in the natural habitat, in agreement with findings of previous studies with M-est and L-est *P. microps* populations [46,65].

### 3.1. Lethal Effects of Cadmium (Cd), Microplastics (MPs) and MPs-Cd Mixtures

In M-est juveniles, no mortality was observed under exposure to 0.14 mg/L of MPs alone (Table S2). The percentages of mortality in treatments containing Cd ranged from 22% to 100% and were similar in corresponding treatments containing Cd alone or in mixture with MPs (Table S2). In L-est juveniles, the treatment containing MPs alone (0.14 mg/L) induced 22% of mortality (Table S2). The percentages of mortality observed in treatments containing Cd alone ranged from 33% to 100% and those recorded in MPs-Cd mixtures ranged from 44% to 100% (Table S2). The integrated analysis of Cd-induced mortality (arcsine transformed percentages of mortality) by two-way ANCOVA indicated no significant differences between M-est and L-est juveniles (F_1, 19_ = 0.029, *p* = 0.866, partial eta squared = 0.002), no significant differences between treatments with and without MPs (ANCOVA = F_1, 19_ = 1.283, *p* = 0.271, partial eta squared = 0.063), and no significant interaction between the two factors (F_1, 19_ = 1.283, *p* = 0.271, partial eta squared = 0.063). These findings indicate that M-est and L-est juveniles have comparable sensitivity to Cd and that MPs did not influence the Cd-induced mortality.

The 96 h LC_10_, LC_20_ and LC_50_ of Cd alone and MPs-Cd mixtures to M-est and L-est juveniles are indicated in Table 1. All the values are comparable, with overlapping 95% CI for corresponding estimates. For example, the 96h-LC_50_ of Cd to M-est juveniles alone or in mixture with MPs was 7 mg/L (95% CI: 2–17 mg/L), whereas the corresponding value of Cd alone was 9 mg/L (95% CI: 0–54 mg/L), and the 96h-LC_50_ of MPs-Cd mixtures was 5 mg/L (95% CI: 0–14 mg/L). These findings also suggest comparable mortality induced by Cd in juveniles from distinct estuaries either alone or in mixture with 0.14 mg/L of MPs.

The total 96h-LC_50_ of Cd alone to *P. microps* juveniles estimated in the present study (8 mg/L, 95% CI: 2–17 mg/L, Table 1) is inside the interval of Cd 96h-LC_50_ values determined to other marine fish that can be found in the literature, ranging from 3.94 mg/L (95% CI: 3.30–4.68 mg/L) to the European sea bass *Dicentrarchus labrax* [85] to 20.12 ± 0.62 mg/L to the white seabass *Lates calcarifer* [86]. Moreover, it is close to the 72h-LC_50_ of 15.32 mg/L estimated to *Sparus aurata* [87].

### 3.2. Effects of Cadmium (Cd), Microplastics (MPs) and MPs-Cd Mixtures on Biomarkers

**Figure 1:**
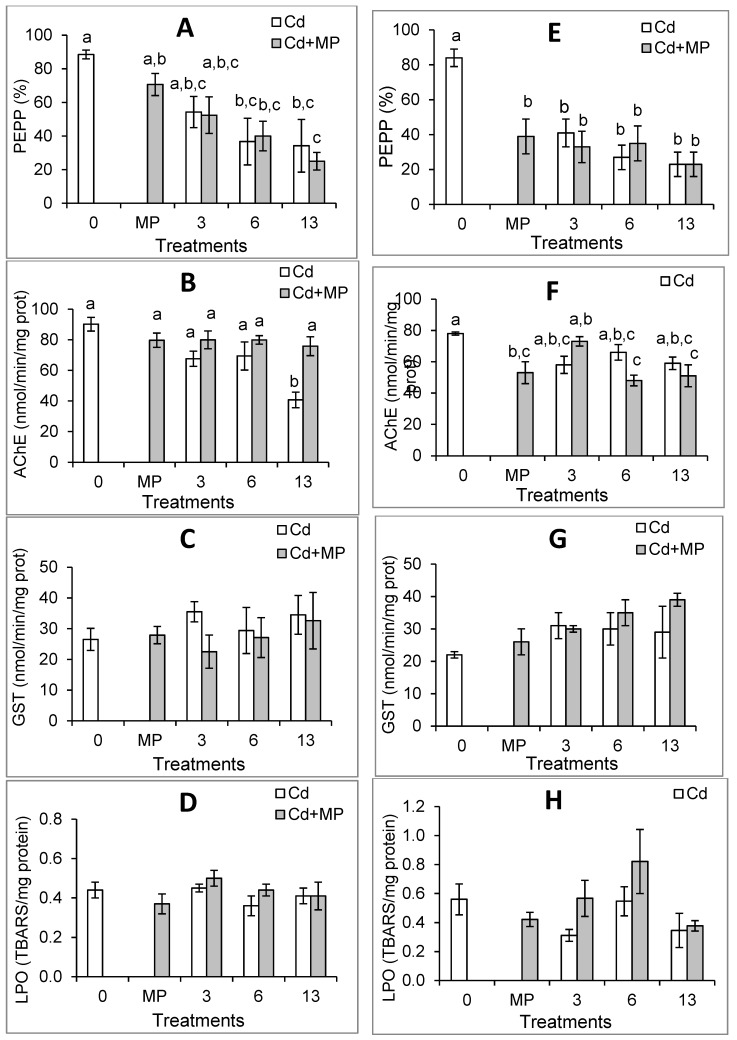
**Mean** and standard error (*N* = 3–9 per treatment) of predatory performance, acetylcholinesterase activity and lipid peroxidation levels *Pomatoschistus microps* juveniles from the estuaries of Minho (A–D) and Lima (E-H) Rivers exposed to cadmium and microplastics individually and in mixture. A and E—predatory performance. B and F—Acetylcholinesterase activity. C and G—Glutathione *S*-transferases activity. D and H—Lipid peroxidation levels. 0—control. MP—treatment containing 0.14 mg/L of microplastics only. 3, 6, 13—treatments containing 3, 6 and 13 mg/L of cadmium (alone or in mixture with microplastics). Different letters above the bars indicate statistically significant differences among treatments (Tukey’s test, *p* ≤ 0.05).

The results of biomarkers are shown in Figure 1. The analysis of each biomarker total data set by 3-ANOVA indicated significant differences (*p* ≤ 0.05) in the predatory performance and in the AChE activity between juveniles from distinct estuaries, as well as some significant (*p* ≤ 0.05) interactions between and/or among fixed factors (Table S3). LPO levels were not significantly different (*p* > 0.05) in juveniles from different estuaries but significant (*p* ≤ 0.05) interaction between estuary and Cd, and between Cd and MPs were found. Thus, the data of these biomarkers were further analyzed for each estuary separately. Regarding GST activity, no significant differences (*p* > 0.05) between juveniles from distinct estuaries, no significant (*p* > 0.05) differences among treatments with distinct Cd concentrations, or among treatments with and without MPs were found (Table S3), thus no further statistical analyses were made.

#### 3.2.1. Effects of Cd, MPs and MPs-Cd Mixtures on Biomarkers of M-Est Juveniles

The results of the analysis of each biomarker data set of M-est juveniles by 2-ANOVA are indicated in Table 2. Significant interaction between Cd and MPs was found for AChE activity, suggesting toxicological interactions between Cd and MPs affecting the activity of this enzyme. For the other parameters, no significant interaction between Cd and MPs was found (Table 2).

To further investigate the effects caused by the substances alone and in mixtures on M-est juveniles, all the individual treatments were compared (Figure 1). Exposure to Cd alone (≥6 mg/L) significantly decreased (by 39–61%) the predatory performance of juveniles (Figure 1A), indicating reduction of their individual fitness. Low food intake likely results in less energy available to face stress (e.g., chemical exposure, diseases, parasite infestations), delays growth and reproduction, and can cause death [46]. Thus, if a considerable number of individuals are affected in the wild, the population fitness may rapidly decrease.

As shown in Figure 1B, exposure to Cd alone also inhibited AChE activity (up to 54%) with significant differences from the control group in fish exposed to 13 mg/L of Cd. Thus, Cd reached the brain and caused neurotoxicity through disruption of cholinergic neurotransmission. Because in teleost fish cholinergic synapses exist both in the brain and muscles, AChE inhibition may cause a wide range of adverse effects, such as visual difficulties, cognitive impairment, deficient movement coordination, swimming, and other behavioral alterations [20,41,46,47,88]. The integrity of the mentioned functions are crucial for the survival of visual predator fish, such as *P. microps*, because they need to locate potential preys at distance, to recognize and discriminate adequate prey from other organisms and particles, to pursue and capture them rapidly [64]. Proper neurologic and neuromuscular functions are also needed to escape from predators and to a high number of physiological functions [46]. Moreover, the intoxication induced by 54% of AChE inhibition may cause reduced appetite and gastrointestinal dysfunction due to the inhibition of the enzyme in muscles of the digestive system. Therefore, AChE inhibition may have contributed to the decrease of the predatory performance of juveniles exposed to Cd alone. Nevertheless, because in the treatment containing 6 mg/L of Cd the AChE activity inhibition was considerably lower (22%) than in juveniles exposed to 13 mg/L of Cd alone, and the difference in the predatory performance between the two groups was not significant (Figure 1A,B), other mechanisms of toxicity must have been involved. These may include death of cholinergic neurons [89], neurotoxicity induced by mechanisms other than AChE inhibition, and other types of toxicity. After absorption, Cd is known to interfere with the biological roles of several essential ions, such as calcium, zinc, and magnesium, among others, and to interact with several biological targets resulting in a wide range of toxic effects [7,11,90,91], the most part of them are partially unrevealed. Some of the effects that have been described in fish exposed to Cd, such as histological alterations in several organs [21], disruption of ion balance [92], changes in the expression of genes related with stress responses [87], cytotoxicity [93], among others [16,17,18,94] may have also contributed to the reduction of the predatory performance found in M-est juveniles exposed to Cd alone. Although the inhibitory effect of Cd on AChE activity of several species is well known [95,96], the mechanisms involved are very complex and are not completely understood even in mammals where they have been intensively studied [e.g., 91,97,98]. Among other mechanisms likely contributing to AChE inhibition, Cd-induced oxidative stress is believed to play an important role [91,97]. In M-est juveniles exposed to Cd, the GST activity was slightly increased but not significantly different from the control group (Figure 1C). No significant changes in LPO levels were found (Figure 1D), thus no significant lipid oxidative damage occurred. Therefore, although these findings cannot exclude a small contribution of oxidative stress to the AChE activity inhibition in M-est juveniles exposed to 13 mg/L of Cd alone, likely this was not the main mechanism involved. An in vitro study with rat brain AChE showed that Cd acts as a non-competitive inhibitor of AChE suggesting that Cd binds to the enzyme in site(s) other than the active one [98]. Thus, it is possible that the inhibition found in M-est juveniles resulted at least partially from Cd binding to AChE. At least in some fish, Cd is also able to inhibit *ache* gene expression and activity in the first hours of exposure, an effect that may be due erroneous coding [99]. Thus, such effect may have also contributed to the AChE inhibition found in M-est juveniles.

The AChE activity inhibition caused by Cd alone found in the present study is in agreement with the anticholinesterase effects of this metal in several other fish species, such as the carps *Labeo rohita*, *Catla catla,* and *Cirrhinus mrigala* [100], the zebra fish *Danio rerio* [20,73,88], the rosy barb *Barbus conchonius* [101], the gilthead seabream *Sparus aurata* [102], the greater amberjack *Seriola dumerili* [103], and the peacock blennie *Salaria pavo* [99], among others [15,96]. However, no significant effects and increased AChE activity in fish exposed to Cd were also reported, sometimes in the same study where inhibition was found but in a different tissue or at a different exposure time [e.g., 101,103]. A recent study with *D. rerio* [88] showed time differences of AChE inhibition induced by Cd among distinct tissues. Therefore, this factor, as well as the time of fish exposure to Cd, the concentrations of Cd tested, the type of cholinesterases present in the species and tissue analyzed, among other factors, may contribute to the apparently distinct findings reported in the literature.

M-est juveniles exposed to Cd alone did not show significant increment of LPO levels, indicating that 96 h of exposure to Cd concentrations up to 13 mg/L did not cause significant lipid oxidative damage. These findings are in good agreement with no significant lipid oxidative damage in the discus fish (*Symphysodon aequifasciatus*) after 30 days of exposure to 0.05 mg/L of Cd [58]. However, Cd-induced oxidative stress and damage in other fish species was documented [16,18,19,93]. Differences in species sensitivity, tissue analyzed, exposure conditions (e.g., cadmium concentrations, exposure time, in vivo or in vitro exposure, route of exposure, test medium), among other factors may have been responsible for such apparently opposite findings.

Regarding the effects of MPs alone, M-est juveniles exposed to 0.14 mg/L of MPs alone had reductions of 20% in the mean predatory performance and of 11% in the mean AChE activity, in both cases with no significant differences in relation to the control group (Figure 1A,B). No significant alterations of LPO levels were also found (Figure 1D). These findings agree with previous studies testing the effects of the same type of MPs on M-est fish under comparable abiotic conditions, where small reductions of the predatory performance and AChE activity in the same order of magnitude (with or without significant differences relatively to the control group), and no significant alterations in LPO levels were reported [38,46,63].

Exposure of M-est juveniles to MPs-Cd mixtures significantly decreased the predatory performance (by 41–72% at Cd concentrations ≥6 mg/L) and no significant differences in relation to juveniles exposed to the corresponding concentrations of Cd alone were found (Figure 1A). These findings and the no significant interaction between MPs and Cd in the predatory performance of M-est fish (Table 2), suggest that Cd was the mainly responsible for the inhibition of the predatory performance in M-est juveniles exposed to MPs-Cd mixtures. Such mixtures did not cause significant effects on AChE activity of M-est juveniles (Figure 1B), despite a slight reduction of the enzymatic activity (11–16%). Therefore, the higher total mean of AChE activity obtained in 2-ANOVA results for juveniles exposed to MPs (Table 2) was mainly due to the lack of significant AChE inhibition in juveniles exposed to the MPs-Cd mixture containing 13 mg/L of Cd. Such results and the significant interaction between Cd and MPs found in 2-ANOVA (Table 2) indicate that MPs antagonized the anticholinesterase effects of Cd in M-est juveniles exposed to the MPs-Cd mixture containing 13 mg/L of Cd. At least three hypotheses (not mutually exclusive) may be raised to explain the antagonism between Cd and MPs in the AChE activity of M-est juveniles exposed to the MPs-Cd mixture containing 13 mg/L of Cd. The first hypothesis is that juveniles exposed to the MPs-Cd mixture may have uptake less Cd from test medium than those exposed to Cd alone, due to the potential adsorption of the metal to MPs in test medium and sedimentation of part of particles decreasing their bioavailability to juveniles. Cd adsorbs to polyethylene MPs [55,56] and the concentrations of MPs in test medium decreased overtime (~34%, Table S1). Although other processes such as the potential uptake of MPs by fish, adsorption of MPs to the internal glass wall of the beakers, among other processes as discussed in [46] may have contributed to the decay of MPs concentrations in test medium, this first hypothesis may explain at least partially the results obtained. The second hypothesis is that in juveniles exposed to the MPs-Cd mixture containing 13 mg/L of Cd part of the metal may have been bound to MPs (adsorption of Cd to MPs may have occurred in test medium and/or inside the body of juveniles) and may have been eliminated from the fish body (e.g., through the gastrointestinal tract) together with MPs without absorption. The decrease of Cd bioaccumulation caused by MPs in fish after chronic exposure [58] provide some support to the potential reduced absorption and/or increased excretion of Cd by *P. microps* in the presence of MPs. However, if occurred, these two processes should have been of small magnitude because juveniles exposed to the Cd-MPs mixture containing 13 mg/L of Cd had significantly lower predatory performance than those exposed to MPs alone but not significantly different from fish exposed to the same concentration of Cd alone (Figure 1A). Therefore, it is possible that absorption of both Cd and MPs occurred resulting in toxic effects, as also suggested by the decrease of MPs concentration in test medium. A third hypothesis is that after potential absorption of both substances somehow the presence of MPs decreased the anticholinesterase effects of Cd. This could happen, for example, if part of Cd is bound to MPs and the MPs-Cd complex cannot reach the enzyme or do not have the capability of interact with it, resulting in decreased anticholinesterase effects. More specific studies would be needed to investigate these hypotheses.

In M-est fish, the most sensitive parameter to Cd and to MPs-Cd mixtures was the predatory performance, LOEC = 6 mg/L (Table 2).

#### 3.2.2. Effects of Cd, MPs, and MPs-Cd Mixtures on Biomarkers of L-Est Juveniles

The results of the 2-ANOVA carried with each biomarker data set of L-est juveniles are shown in Table 2. Significant interaction between MPs and Cd in the predatory performance and AChE activity was found, suggesting toxicological interactions between the two substances affecting these parameters. No significant interaction in LPO levels was found.

L-est juveniles exposed to all treatments containing Cd alone had significantly decreased (by 51–73%) predatory performance in relation to the control group (Figure 1E), indicating reduction of individual fitness. Based on the predatory performance, L-est juveniles (LOEC = 3 mg/L) were more sensitive to Cd alone than M-est juveniles (LOEC = 6 mg/L). Thus, the environmental conditions (e.g., pollution, food availability) of the population habitat and exposure to such conditions in pre-developmental phases influence the predatory performance of fish under Cd sub-lethal acute stress.

Contrary to M-est juveniles, L-est juveniles exposed to Cd alone did not show significant AChE activity inhibition in relation to the respective control group (Figure 1F). Part of this may be due to the lower mean of AChE activity in the L-est control group than the corresponding mean of the M-est control group. However, this does not seem to be enough to completely explain the difference between juveniles of distinct estuaries exposed to the highest concentration of Cd. One can raise the hypothesis that because the L-est estuary has higher levels of some metals in sediments, including Cd, than those found in the M-est [65], the L-est population developed mechanisms decreasing the amount of Cd able to reach and interact with AChE. The Cd in circulation was enough to significantly decrease the predatory performance of L-est juveniles (Figure 1E). Therefore, although not totally excluding the possibility of decreased Cd uptake and/or increased elimination in L-est juveniles, these findings suggest that other mechanisms may have been involved. A common response of fish to Cd exposure is the increase of certain proteins, including metallothioneins, and enzymes, and long-term induction of such endogenous molecules contributes to Cd tolerance [104]. Therefore, one possibility is that a slight increase of such endogenous molecules binding Cd occurred in L-est. If so, it may have been sufficient to protect AChE but not high enough to avoid other toxic effects contributing to predatory performance reduction.

As in M-est juveniles, no significant differences in LPO levels were found in L-est juveniles (Figure 1G,H). Although with no significant differences in relation to the control group, GST activity was induced (~1.4 folds) suggesting that oxidative stress may have occurred and have been overcome through the activation of the antioxidant system preventing lipid oxidative damage to occur (Figure 1H).

L-est fish exposed to MPs alone had significantly decreased (54%) predatory performance (Figure 1E), indicating significant reduction of their fitness, as previously discussed (Section 3.2). In the experimental conditions used (i.e., very small MPs, no food, and no apparent skin damage), fish likely ingested MPs together with test medium entering passively through the mouth and may have also uptake MPs through the gills during respiration. As reported in several studies with fish and other organisms, MPs in the digestive system can cause false food satiation [70]. Therefore, one hypothesis that can explain the reduced predatory performance of L-est juveniles is the potential presence of MPs in the gastrointestinal tract causing false food satiation leading to decreased search for food. As L-est fish also had significant AChE activity inhibition (32%, Figure 1F), another hypothesis is that the inhibition of this enzyme may have contributed to the decrease of the predatory performance, for example through the impairment of brain and/or muscular functioning as previously discussed for Cd. Additionally, other effects that have been described in fish exposed to MPs, such as MPs in gills making respiration difficult, lesions in the gastrointestinal system caused by the particles, alterations in the energy metabolism, among others [42,43,44,48,58] may have also occurred and if so they may have contributed to the predatory performance decrease. The MPs-induced reduction of L-est juveniles predatory performance is in agreement with previous findings in L-est fish exposed to the same type of MPs [46], and with the reduced predatory performance of *P. microps* juveniles [64] and of the Amazonian cichlid *Symphysodon aequifasciatus* [42] exposed to other types of MPs. It also agrees with the decreased swimming distance in the Crucian carp *Carassius carassius* [105], with the decreased swimming velocity in *D. labrax* [47], and with the decreased swimming behavior in the sheepshead minnow *Cyprinodon variegatus* [48] exposed to other types of MPs (micro or nanosized).

The significant AChE activity inhibition (32%) in L-est exposed to microplastics (Figure 1F) indicates neurotoxicity and agrees with the anticholinesterase effects of MPs previously found in L-est *P. microps* juveniles [46], other fish species [39,41,42], and other organisms [106]. MPs of several sizes, including some much larger than those tested here (in some cases larger than 300 µm) were found in internal organs (e.g., liver) and tissues (e.g., dorsal muscle) of wild fish [35,36,107], indicating that somehow they crossed biological barriers and entered into the blood stream. Moreover, in experimental studies, MPs were found in the brain of the Crucian carp *Carassius carassius* [105] exposed to 0.053 µm or to 0.180 µm particles and of the red tilapia (*Oreochromis niloticus*) exposed to 0.1 µm MPs [39] indicating that the particles crossed the blood-brain barrier and entered into the brain. Moreover, *C. carassius* exposed to nano-sized MPs also showed behavior alterations suggesting that such alterations may have been due to the presence of the particles in the brain [105]. Furthermore, *O. niloticus* exposed to MPs also had brain AChE activity inhibition suggesting an association between the presence of the particles and the inhibition of the enzyme [39].

L-est fish exposed to all the MPs-Cd mixtures had significantly decreased predatory performance (61–73% at concentrations ≥3 mg/L) with no significant differences in relation to corresponding concentrations of Cd and MPs alone (Figure 1E). Thus, the effects of MPs-Cd mixtures were lower than the sum of the effects caused by MPs alone (54% inhibition) and by Cd alone (51–73% inhibition). Mixtures containing Cd at concentrations ≥6 mg/L also caused significant AChE inhibition (Figure 1F), namely 15% and 24%. The same concentrations Cd alone caused no significant inhibition of AChE activity despite a decrease of the enzymatic activity in relation to the control group. MPs alone caused 32% of AChE inhibition. Such results and the significant interactions found by 2-ANOVA in both predatory performance and AChE activity (Table 2) suggest antagonism between MPs and Cd on the predatory performance and AChE activity of L-est fish exposed to MPs-Cd mixtures. Regarding GST activity and LPO levels in L-est fish exposed to MPs-Cd mixtures, despite the lack of significant differences, the higher GST activity means (1.4–1.8 folds) in relation to the control group, and the increasing trend with the increase of the Cd concentration in the mixtures (1.4–1.8 folds) suggest that mixtures may have caused low levels of stress in L-est fish leading to the activation of antioxidant defenses preventing significant lipid oxidative damage to occur.

The toxicological interactions between Cd and MPs (polystyrene) were previously investigated in chronic studies with two freshwater fish. In *Symphysodon aequifasciatus*, MPs (32–40 µm) reduced Cd bioaccumulation, antagonized the effects of Cd in some parameters (e.g., acid phosphatase activity, alkaline phosphatase activity) but the mixtures caused higher oxidative stress and other toxic effects than Cd or MPs individually [42]. In *D. rerio,* MPs (5 µm) increased the bioaccumulation and toxicity of Cd, and mixtures induced oxidative stress [57]. The comparison of the findings in *S. aequifasciatus* [42], *D. rerio* [57] and in *P. microps* from M-est and L-est (Figure 1) show that the properties of MPs, the species and original population of the fish, exposure time, and concentrations of the pollutants tested, among other factors may influence the toxicity of MPs, Cd, and their mixtures.

In L-est fish, the most sensitive parameter to Cd and MPs-Cd mixtures was the predatory performance (LOEC = 3 mg/L), as shown in Table 2.

### 3.3. Implications to Environmental and Human Risk Assessment

The results of the present study showed reduction of the predatory performance and neurotoxicity in *P. microps* juveniles exposed to Cd, MPs, and MPs-Cd mixtures. Intoxicated fish generally consume less food and are more susceptible to diseases and parasite infestations. Thus, such fish likely have a decreased health status in relation to healthy fish and contribute less to population sustainability. At long term, this may lead to population reduction and less prey available to higher predators with adverse consequences to higher trophic levels, to ecosystem functioning and the services provided to the human society, including human food security [4]. Moreover, Cd and MPs are accumulated by wild fish [12,13,35]. Intoxicated fish are more prone to be captured by predators that generally ingest a high number of preys potentially leading to their contamination by considerable amounts of Cd, MPs and the chemicals that MPs may contain. Predators of *P. microps* include species (e.g., higher trophic level fish, shrimps) widely consumed as food by humans. Therefore, humans consuming *P. microps* predators from ecosystems polluted with Cd and MPs may be at increased risk of exposure to these substances, and to the chemicals and microorganisms that MPs may have.

Fish and other aquatic animals from different regions, including from aquaculture, consumed as food by humans have been found to be contaminated by MPs [32,37,107,108,109]. Although the studies are still limited, estimates of human exposure from fish and shellfish intake are available in the literature [36,110]. Fish and other aquatic animals from polluted sites likely have higher amounts of MPs in edible tissues than those from less contaminated areas. Other human food items have been also found to be contaminated by MPs [4,111], and data is not available for several others (e.g., terrestrial animals and vegetable food sources). Moreover, several other exposure routes of humans to MPs exist, such as drinking water and air [4,5,31,112]. A recent study documented the presence of MPs in human stool [113] (Schwabl et al., 2018), demonstrating that humans intake MPs and eliminate at least some of them. Likely, absorption of small MPs occurs, as happens in animals such as fish. Thus, more research on human exposure to MPs by different routes, absorption, and elimination from the human body is needed.

Studies in animals showed that nano-sized MPs can reach the brain [39,105]. Moreover, MPs can cause neurotoxicity [38,39,41,46,105], impair reproduction [114,115] and cause transgenerational effects [116], among other types of toxicity [50,51,106,117]. Cytotoxicity was found in cerebral and epithelial human cells [118] and adverse effects of occupational exposure to MPs are documented [112]. Thus, high concern regarding environmental, animal, and human health effects resulting from exposure to MPs exists.

Despite the amount of studies investigating the effects of MPs carried out in the last decades, only a limited number of MPs types were investigated, whereas the MPs released to the environment include a very high diversity of particles whose properties change considerably during their long permanence in the environment [25]. Moreover, MPs contain additives and other chemicals incorporated during the production of plastic products and their use, respectively, as well as environmental contaminants and microorganisms adsorbed to the particles in the environment [4,25,30,31]. The most part of such chemicals are also very toxic and microorganisms may include pathogens [4,31]. Furthermore, in real scenarios, organisms and humans are exposed simultaneously and during their entire life to MPs and several other environmental contaminants and toxicological interactions may occur. Indeed, several laboratory studies documented toxicological interactions between MPs and other environmental contaminants in animals, including polycyclic aromatic hydrocarbons [38,119,120], polychlorinated biphenyls and brominated flame retardants [121], pharmaceuticals [63], chromium [46], silver [122], nickel [123], gold nanoparticles [115], mercury [40,41,47], cadmium [57,58], sometimes increasing chemical toxicity, and bioaccumulation [40,57]. Therefore, more research on these topics is urgently needed to improve risk assessment and environmental and human safety.

The present study showed some differences in the toxic effects induced by Cd, MPs, and their mixtures between two populations of *P. microps*, in good agreement with a previous study investigating the effects of MPs, Cr, and their mixtures [46]. Inter-population variability in the susceptibility to chemicals is common in other animals and humans. Typically, the hazard assessment of chemicals for environmental and human risk assessment purposes is done with laboratory organisms of a single population, often with a limited genetic variability. As the uptake, toxicity, and elimination of chemicals may be different among distinct populations, an additional challenge regarding human and environmental risk assessment of MPs and associated chemicals is to take into consideration inter-population variability in a more effective way.

Temperature and other abiotic factors are able to modulate the toxicity and bioaccumulation of MPs, several other pollutants, and mixtures. For example, temperature rise can increase MPs toxicity and bioaccumulation [42] and change the way how MPs influence the toxicity of other environmental contaminants [63]. Therefore, the modulation of MPs and their mixtures with other chemicals by abiotic factors is of most interest to improve the adaptation of the human society to climate changes. The type of modulation often changes with the properties of tested substances, biological factors (e.g., sex, age, nutritional status, and physical condition), and environmental conditions. Therefore, extrapolations and predictions are challenging and the data available is still limited. Generally, for environmental and human risk assessment purposes, the toxicity evaluations are done at one single and fixed temperature, with other fixed environmental factors to reduce experimental variability. Considering the global warming and increased environmental variability already in course due to global climate changes, more studies regarding the effects of temperature and other environmental conditions (e.g., air humidity, water salinity, pH) variation on the uptake, biotransformation, accumulation, toxicity, and elimination of MPs, associated chemicals, and mixtures are needed to improve environmental and human risk assessment of MPs and other environmental contaminants.

## 4. Conclusions

Cd alone caused comparable lethal effects on M-est and L-est juveniles, with overall 96h-LC_10_ of 2 mg/L (95% CI: 0–4 mg/L) and 96h-LC_50_ of 8 mg/L (95% CI: 2–17 mg/L). No significant differences in the mortality induced by Cd and by MPs-Cd mixtures were found. Therefore, MPs (0.14 mg/L) did not modulate the Cd-induced mortality in *P. microps* juveniles.

Exposure (96 h) of *P. microps* juveniles to sub-lethal concentrations of Cd significantly decreased the predatory performance (M-est: NOEC = 3 mg/L, LOEC = 6 mg/L; L-est: NOEC < 3 mg/L, LOEC = 3 mg/L) and significantly inhibited AChE activity in M-est juveniles (NOEC = 6 mg/L, LOEC = 13 mg/L), but not in L-est juveniles (NOEC = 13 mg/L). MPs alone (0.14 mg/L) significantly inhibited AChE activity and the predatory performance of L-est juveniles but not of M-est juveniles. MPs-Cd mixtures significantly decreased the predatory performance of juveniles from both estuaries (M-est: NOEC = 3 mg/L, LOEC = 6 mg/L; L-est: NOEC < 3 mg/L, LOEC = 3 mg/L), and caused significant neurotoxicity in L-est juveniles (NOEC = 3 mg/L, LOEC = 6 mg/L) but not in M-est fish (NOEC = 13 mg/L). Thus, Cd, MPs, and MPs-Cd mixtures caused neurotoxicity and reduced the individual fitness of *P. microps* juveniles. Evidences of toxicological interactions between Cd and MPs in the predatory performance and AChE activity of *P. microps* juvenis were found, namely antagonism. Differences in the sub-lethal effects induced by Cd, MPs, and MPs-Cd mixtures on *P. microps* juveniles from distinct estuaries were observed. Inter-population variability in the sensitivity to pollutants and other environmental stressors (e.g., temperature) is common in other species, including humans. More knowledge on the effects of MPs (alone and in mixture with other environmental contaminants), on the modulation of chemical toxicity by temperature and other environmental variables, and on inter-population variability in susceptibility to MPs and other chemicals is needed.

In response to findings resulting from the research that has been carried in the last decades and to public preoccupation regarding plastic and MPs pollution and its effects, important policies, regulations, and initiatives have been implemented, such as the monitoring of marine litter (including MPs) in marine waters of the European Union (EU) in the scope of the Marine Strategy Framework Directive (Directive 2008/56/EC), the EU Plastics Strategy, and more recently the European Chemicals Agency restriction dossier on MPs added in products that is in public consultation. Also of high importance is to assess and manage the risk of human exposure to MPs. Therefore, more knowledge on the potential effects of MPs and associated chemicals on human health, environmental and human food quality, and human exposure are needed to increase human and environmental safety under the current scenario of global pollution by MPs and climate changes.

## Figures and Tables

**Table 1:** Estimated 96 h lethal concentrations (LC_10_, LC_20_ and LC_50_ of cadmium alone (Cd) and in mixture with microplastics (Cd + MP) to *Pomatochistus microps* juveniles from the estuaries of Minho and Lima Rivers with corresponding 95% confidence interval within brackets, and results of the Analysis of Covariance for several comparisons are also shown. No observed effect concentrations (NOEC) and lowest observed effect concentrations (LOEC) of Cd and MP alone, and of Cd in mixture with MP (Cd + MP) for biomarkers. Variab. Contrib.—contribution to total variance; PEPP—post-exposure predatory performance; AChE—acetylcholinesterase activity; GST—glutathione *S*-transferases activity; LPO—lipid peroxidation levels.

**Bioassay**	**Minho Estuary Fish**					**Lima Estuary Fish**				
	**LC_10_ (mg/L)**	**LC_20_ (mg/L)**			**LC_50_ (mg/L)**	**LC_10_ (mg/L)**	**LC_20_ (mg/L)**			**LC_50_ (mg/L)**
Cd	2 (0–5)	3 (0–7)			7 (2–17)	2 (0–5)	3 (0–7)			9 (0–54)
Cd + MP	2 (0–5)	3 (0–7)			7 (2–17)	2 (0–4)	2 (0–5)			5 (0–14)
**Overall: Fish from Both Estuaries**										
Cd						Cd + MP				
LC_10_ (mg/L)	LC_20_ (mg/L)		LC_50_ (mg/L)			LC_10_ (mg/L)	LC_20_ (mg/L)		LC_50_ (mg/L)	
2 (0–4)	3 (0–6)		8 (2–17)			2 (0–4)	3 (0–6)		9 (0–15)	
**Biomarkers**										
**Biomarker**		**Contaminants**			**M-Est Fish**			**L-Est Fish**		
					**NOEC (mg/L)**	**LOEC (mg/L)**		**NOEC (mg/L)**		**LOEC (mg/L)**
PEPP		Cd MP Cd + MP			3 0.14 3	6 >0.14 6		<3 <0.14 <3		3 0.14 3
AChE		Cd MP Cd + MP			6 0.14 13	13 >0.14 >13		13 <0.14 3		> 13 0.14 6
GST		Cd MP Cd + MP			13 0.14 13	>13 >0.14 >13		13 0.14 13		>13 >0.14 >13
LPO		Cd MP Cd + MP			13 0.14 13	>13 >0.14 >13		13 0.14 13		>13 >0.14 >13

**Table 2:** Results of two-way ANOVA with interaction (fixed factors: cadmium concentration and microplastics presence) carried out with *Pomatoschistus microps* juveniles from the estuaries of Minho and Lima rivers. BIOM—Biomarker. FAC—Factor. Pred.—predatory performance. AChE—Acetylcholinesterase activity (nmol/min/mg protein). GST—Glutathione *S*-transferases activity (nmol/min/mg protein). LPO—lipid peroxidation levels (TBARS/mg protein). Cd—cadmium concentration. Inter.—interaction. N—number of fish analyzed. SD—Standard deviation. Different letters after the mean indicate statistically significant differences among distinct concentrations of cadmium (*p* ≤ 0.05, Tukey’s test).

BIOM	FAC	Level	Minho Estuary Fish			Lima Estuary Fish		
			*N*	Mean ± SD	ANOVA Results	*N*	Mean ± SD	ANOVA Results
Pred.	Cd	0 mg/L	18	80 ± 17 a	F_3, 42_ = 14.146, *p* < 0.001	16	64 ± 30 a	F_3, 38_ = 10.124, *p* < 0.001
		3 mg/L	14	53 ± 26 b		11	37 ± 19 b	
		6 mg/L	10	38 ± 25 b		11	31 ± 20 b	
		13 mg/L	8	30 ± 22 b		8	23 ± 13 b	
	MP	No	25	60 ± 32	F_1, 42_ = 1.413, *p* = 0.241	26	49 ± 31	F_1, 38_ = 4.873, *p* = 0.033
		Yes	25	52 ± 27		20	34 ± 21	
	Inter.				F_3, 42_ = 1.011, *p* = 0.397			F_3, 38_ = 6.253, *p* = 0.001
AChE	Cd	0 mg/L	18	85 ± 14 a	F_3, 42_ = 6.956, *p* = 0.001	16	67 ± 17	F_3, 38_ = 3.281, *p* = 0.031
		3 mg/L	14	74 ± 15 a,b		11	65 ± 13	
		6 mg/L	10	75 ± 15 a		11	58 ± 14	
		13 mg/L	8	58 ± 22 b		8	56 ± 11	
	MP	No	25	72 ± 22	F_1, 42_ = 8.237, *p* = 0.006	26	67 ± 12	F_1, 38_ = 7.170, *p* = 0.011
		Yes	25	79 ± 12		20	56 ± 15	
	Inter.				F_3, 42_ = 5.301, *p* = 0.003			F_3, 38_ = 8.221, *p* < 0.001
LPO	Cd	0 mg/L	18	0.4 ± 0.1	F_3, 42_ = 1.536, *p* = 0.219	16	0.5 ± 0.3	F_3, 38_ = 2.482, *p* = 0.076
		3 mg/L	14	0.5 ± 0.1		11	0.4 ± 0.2	
		6 mg/L	10	0.4 ± 0.1		11	0.7 ± 0.4	
		13 mg/L	8	0.4 ± 0.1		8	0.4 ± 0.2	
	MP	No	25	0.4 ± 0.1	F_1, 42_ = 0.299, *p* = 0.587	26	0.5 ± 0.3	F_1, 38_ = 1.582, *p* = 0.216
		Yes	25	0.4 ± 0.1		20	0.6 ± 0.3	
	Inter.				F_3, 42_ = 1.280, *p* = 0.293			F_3, 38_ = 1.690, *p* = 0.185

## Supplementary Materials

The following are available online at https://www.mdpi.com/1660-4601/16/16/2857/s1, Table S1: Nominal and actual concentrations of microplastics in freshly prepared (0 h) and old test medium (96 h) of different treatments of the bioassays carried out with fish from the estuaries of Minho (M-est) and Lima (L-est) fish. The results are the mean (*n* = 3 per treatment) with corresponding standard deviation. The results of the two-way ANOVA carried with each variable to investigate differences in microplastics concentrations among treatments and between bioassays are also indicated. FLUO 0 h—fluorescence at the beginning of the bioassay. CONC 0 h—concentration of microplastics at the beginning of the bioassay. FLUO 96 h—fluorescence at the end of the bioassay. CONC 96 h—concentration of microplastics at the end of the bioassay. DECAY—percentage of decrease of microplastics concentrations. EEC—estimated exposure concentration during the bioassay. Table S2: Mortality recorded in fish from the estuaries of Minho (M-est) and Lima (L-est) Rivers in the bioassays testing the effects of cadmium, microplastics and their mixtures. N1—number of individuals per treatment at the beginning of the bioassay. N2—number of dead fish from the M-est. N3—number of dead fish from the L-est. Mort—percentage of mortality. MP—microplastics (0.14 mg/l). 3 Cd—treatment with 3 mg/l of cadmium. 3 Cd + MP – mixture containing 3 mg/l of cadmium + 0.14 mg/l of MP. 6 Cd – treatment with 6 mg/l of cadmium. 6 Cd + MP – mixture containing 6 mg/l of cadmium + 0.14 mg/l of MP. 13 Cd – treatment with 13 mg/l of cadmium. 13 Cd + MP – mixture containing 13 mg/l of cadmium + 0.14 mg/l of MP. Table S3: Results of three-way ANOVA (3-ANOVA) with interactions (fixed factors: estuary, cadmium concentration and microplastics presence) carried out with *Pomatoschistus microps* juveniles from the estuaries of Minho and Lima rivers. Predatory performance—post-exposure predatory performance. AChE activity—Acetylcholinesterase activity. GST activity—Glutathione *S*-transferases activity. LPO levels—lipid peroxidation levels. Est—original estuary of the fish. Cd—cadmium concentration. MP—presence/absence of microplastics. Est × Cd—interaction between estuary and cadmium concentration. Est × MP—interaction between estuary and microplastics. Cd × MP—interaction between cadmium concentration and microplatics. Cd × MP—interaction between cadmium concentration and microplatics. Est × Cd × MP—interaction among estuary, cadmium and microplastics.

## References

[1] WHO (2010). Preventing Disease through Healthy Environments. Exposure to Cadmium: A Major Public Health Concern.

[2] Ng E.-L., Lwanga E.H., Eldridge S.M., Johnston P., Hu H.-W., Geissen V., Chen D. (2018). An overview of microplastic and nanoplastic pollution in agroecosystems. Sci. Total Environ..

[3] Horton A.A., Walton A., Spurgeon D.J., Lahive E., Scendsen C. (2017). Microplastics in freshwater and terrestrial environments: Evaluating the current understanding to identify the knowledge gaps and future research priorities. Sci. Total Environ..

[4] Barboza L.G.A., Vethaak A.D., Lavorante B.R.B.O., Lundebye A.-K., Guilhermino L. (2018). Marine microplastics debris: An emerging issue for food security, food safety and human health. Mar. Pollut. Bull..

[5] Lehner R., Weder C., Petri-Fink A., Rothen-Rutishauser B. (2019). Emergence of nanoplastic in the environment and possible impact on human health. Environ. Sci. Technol..

[6] Nascimento S.F., Kurzweill H., Wruss W., Fenzl N. (2006). Cadmium in the Amazonian Guajará estuary: Distribution and remobilization. Environ. Pollut..

[7] Matović V., Buha A., Bulat Z., Đurić-Ćosić D., Bulat Z. (2015). Inside into the oxidative stress induced by lead and/or cadmium in blood, liver and kidneys. Food Chem. Toxicol..

[8] European Commission (2013). Directive 2013/39/EU of the European parliament and of the council of 12 August 2013 amending directives 200/60/EC and 2008/105/EC as regards priority substances in the field of water policy. J. Eur. Union.

[9] US EPA (2016). Recommended Aquatic Life Ambient Water Quality Criteria for Cadmium.

[10] International Agency for Research on Cancer (IARC) (1993). Cadmium and cadmium compounds. IARC Monographs on the Evaluation of Carcinogenic Risks to Humans. Vol. 58. Beryllium, Cadmium, Mercury, and Exposures in the Glass Manufacturing Industry.

[11] Matović V., Buha A., Bulat Z., Đurić-Ćosić D. (2011). Cadmium toxicity revisited: Focus on oxidative stress induction and interactions with zinc and magnesium. Arth. Hig. Rada Toksikol..

[12] Caçador I., Costa J.L., Duarte B., Silva G., Medeiros J.P., Azeda C., Castro N., Freitas J., Pedro S., Almeida P.R. (2012). Macroinvertebrates and fishes as biomonitors of heavy metal concentration in the Seixal Bay (Tagus estuary): Which species perform better?. Ecol. Indic..

[13] Bosch A.C., O’Neill B., Sigge G.O., Kerwath S.E., Hoffman L.C. (2016). Heavy metals in marine fish meat and consumer health: A review. J. Sci. Food Agric..

[14] Zhao X.-M., Yao L.-A., Ma Q.-L., Zhou G.-J., Wang L., Fang Q.-L. (2018). Distribution and ecological risk assessment of cadmium in water and sediment in Longjiang River, china: Implications on water quality management after pollution accident. Chemosphere.

[15] de Araújo M.C., Assis C.R.D., Silva L.C., Machado D.C., Silva K.C.C., Lima A.V.A., Carvalho L.B., de Souza Bezerra S., Oliveira M.B.M. (2016). Brain acetylcholinesterase of jaguar cichlid (*Parachromis managuensis*): From physicochemical and kinetic properties to its potential as biomarker of pesticides and metal ions. Aquat. Toxicol..

[16] Liu X.-J., Luo Z., Li C.-H., Xiong B.-X., Zhao Y.-H., Li X.-D. (2011). Antioxidant responses, hepatic intermediary metabolism, histology and ultrastructure in *Synechogobius hasta* exposed to waterborne cadmium. Ecotoxicol. Environ. Saf..

[17] Dew W.A., Veldhoen N., Carew A.C., Helbing C.C., Pye G.G. (2016). Cadmium-induced olfactory dysfunction in rainbow trout: Effects of binary and quaternary metal mixtures. Aquat. Toxicol..

[18] Wang C.-C., Si L.-F., Guo S.-N., Zheng J.-L. (2019). Negative effects of acute cadmium on stress defense, immunity, and metal homeostasis in liver of zebrafish: The protective role of environmental zinc pre-exposure. Chemosphere.

[19] Pereira L.S., Ribas J.L.C., Vicari T., Silva S.B., Stival J., Baldan A.P., Valdez Domingos F.X., Grassi M.T., Cestari M.M., Silva de Assis H.C. (2016). Effects of ecologically relevant concentrations of cadmium in a freshwater fish. Ecotoxicol. Environ. Saf..

[20] Pan H., Zhang X., Ren B., Yang H., Ren Z., Wang W. (2017). Toxic assessment of cadmium based online swimming behaviour and the continuous AChE activity in the gill of zebrafish. Water Air Soil Pollut..

[21] Giari L., Manera M., Simoni E., Dezfuli B.S. (2007). Cellular alterations in diferente organs of European sea bass *Dicentrarchus labrax* (L.) exposed to cadmium. Chemosphere.

[22] Hani Y.M.I., Turies C., Palluel O., Delahaut L., Bado-Nilles A., Geffard A., Dedourge-Geffard O., Porcher J.-M. (2019). Effects of a chronic exposure to different water temperatures and/or to an environmental cadmium concentration on the reproduction of the threespine stickleback (*Gasterosteus aculeatus*). Ecotoxicol. Environ. Saf..

[23] Krzykwa J.C., Saeid A., Jeffries M.K.S. (2019). Identifying sublethal endpoints for evaluating neurotoxic compounds utilizing the fish embryo toxicity test. Ecotoxicol. Environ. Saf..

[24] Raimundo J., Vale C., Martins I., Fontes J., Graça G., Caetano M. (2015). Elemental composition of two ecologically contrasting seamount fishes, the bluemouth (*Helicolenus dactylopterus*) and blackspot seabream (*Pagellus bogaraveo*). Mar. Pollut..

[25] Andrady A.L. (2017). The plastic in microplastics. Mar. Pollut. Bull..

[26] Lasee S., Maurício J., Thompson W.A., Karnjanapiboonwong A., Kasumba J., Subbiah S., Morse A.N., Anderson T.A. (2017). Microplastics in a freshwater environment receiving treated wastewater effluent. Integr. Environ. Assess. Manag..

[27] Antunes J., Frias J., Sobral P. (2018). Microplastics in the Portuguese coast. Mar. Pollut. Bull..

[28] Barrows A.P.W., Cathey S.E., Petersen C.W. (2018). Marine environment microfiber contamination: Global patherns and the diversity of microparticle origins. Environ. Pollut..

[29] Hartmann N.B., Rist S., Bodin J., Jensen L.H., Schmidt S.N., Mayer P., Meibom A., Baun A. (2017). Microplastics as vectors for environmental contaminants: Exploring sorption, desorption, and transfer to biota. Integr. Environ. Assess. Manag..

[30] Hahladakis J.N., Velis C.A., Weber R., Lacovidou E., Purnell P. (2018). An overview of chemical additives present in plastics: Migration, release, fate and environmental impact during their use, disposal and recycling. J. Hazard. Mater..

[31] Wright S.L., Kelly J.F. (2017). Plastic and human health: A micro issue?. Environ. Sci. Technol..

[32] Neves D., Sobral P., Ferreira J.L., Pereira T. (2015). Ingestion of microplastics by commercial fish of the Portuguese coast. Mar. Pollut. Bull..

[33] Rochman C.M., Tahir A., Williams S.L., Baxa D.V., Lam R., Miller J.T., Teh F.-C., Werorilangi S., Teh S.J. (2015). Anthropogenic debris in seafood: Plastic debris and fibers from textiles in fish and bivalves sold for human consumption. Sci. Rep..

[34] Bessa F., Barría P., Neto J.M., Frias J.P., Otero V., Sobral P., Marques J.C. (2018). Occurrence of microplastics in commercial fish from a natural estuarine environment. Mar. Pollut. Bull..

[35] Abbasi S., Soltani N., Keshavarzi B., Moore F., Turner A., Hassanaghaei M. (2018). Microplastics in different tissues of fish and prawn from the Musa Estuary, Persian Gulf. Chemosphere.

[36] Akhbarizadeh R., Moore F., Keshavarzi B. (2018). Investigating a probable relationship between microplastics and potentially toxic elements in fish muscles from northeast of Persian Gulf. Environ. Pollut..

[37] Smith M., Love D.C., Rochman C.M., Neff R.A. (2018). Microplastics in seafood and the implications for human health. Curr. Environ. Health Rep..

[38] Oliveira M., Ribeiro A., Hylland K., Guilhermino L. (2013). Single and combined effects of microplastics and pyrene on juveniles (0+ group) of the common goby *Pomatoschistus microps* (Teleostei: Gobiidae). Ecol. Indic..

[39] Ding J., Zhang S., Razanajatovo R.M., Zou H., Zhu W. (2018). Accumulation, tissue distribution, and biochemical effects of polystyrene microplastics in the freshwater fish red tilapia (*Oreochromis niloticus*). Environ. Pollut..

[40] Barboza L.G.A., Vieira L.R., Branco V., Carvalho C., Guilhermino L. (2018). Microplastics increase mercury bioconcentration in gills and bioaccumulation in the liver, and cause oxidative stress and damage in *Dicentrarchus labrax* juveniles. Sci. Rep..

[41] Barboza L.G.A., Vieira L.R., Branco V., Figueiredo N., Carvalho F., Carvalho C., Guilhermino L. (2018). Microplastics cause neurotoxicity, oxidative damage and energy-related changes and interact with the bioaccumulation of mercury in the European seabass *Dicentrarchus labrax* (Linnaeus, 1758). Aquat. Toxicol..

[42] Wen B., Zhang N., Jin S.R., Chen Z.Z., Gao J.Z., Liu Y., Liu H.P., Xu Z. (2018). Microplastics have a more profound impact than elevated temperatures on the predatory performance, digestion and energy metabolism of an Amazonian cichlid. Aquat. Toxicol..

[43] Pedà C., Caccamo L., Fossi M.C., Cai F., Andaloro F., Genovese L., Perdichizzi A., Romeo T., Maricchiolo G. (2016). Intestinal alterations in European sea bass *Dicentrarchus labrax* (Linnaeus, 1758) exposed to microplastics: Preliminary results. Environ. Pollut..

[44] Lei L., Wu S., Lu S., Liu M., Song Y., Fu Z., Shi H., Raley-susman K.M., He D. (2018). Microplastic particles cause intestinal damage and other adverse effect in zebrafish *Danio rerio* and nematode *Caenorhabditis elegans*. Sci. Total Environ..

[45] Yin L., Chen B., Xia B., Shi X., Qu K. (2018). Polystyrene microplastics alter the behavior, energy reserve and nutritional composition of marine jacopever (*Sebastes schlegelii*). J. Hazard. Mater..

[46] Luis L.G., Ferreira P., Fonte E., Oliveira M., Guilhermino L. (2015). Does the presence of microplastics influence the acute toxicity of chromium (VI) to early juveniles of the common goby (*Pomatoschistus microps*)? A study with juveniles from two wild estuarine populations. Aquat. Toxicol..

[47] Barboza L.G.A., Vieira L.R., Guilhermino L. (2018). Single and combined effects of microplastics and mercury on juveniles of the European seabass (*Dicentrarchus labrax*): Changes in behavioural responses and reduction of swimming velocity and resistance time. Environ. Pollut..

[48] Choi J.S., Jung Y.-J., Hong N.-H., Hong S.H., Park J.-W. (2018). Toxicological effects of irregularly shaped and spherical microplastics in a marine teleost, the sheepshead minnow (*Cyprinodon variegatus*). Mar. Pollut. Bull..

[49] Rochman C.M., Kurobe T., Flores I., Teh S.J. (2014). Early warning signs of endocrine disruption in adult fish from the ingestion of polyethylene with and without sorbed chemical pollutants from the marine environment. Sci. Total Environ..

[50] Alimba C.G., Faggio C. (2019). Microplastics in the marine environment: Current trends in environmental pollution and mechanisms of toxicological profile. Environ. Toxicol. Pharmacol..

[51] Franzellitti S., Canesi L., Auguste M., Wathsala R.H.G.R., Fabbri E. (2019). Microplastic exposure and effects in aquatic organisms: A physiological perspective. Environ. Toxicol. Pharmacol..

[52] Guilhermino L., Vieira L.R., Ribeiro D., Tavares A.S., Cardoso V., Alves A., Almeida J.M. (2018). Uptake and effects of the antimicrobial florfenicol, microplastics and their mixtures on freshwater exotic invasive bivalve *Corbicula fluminea*. Sci. Total Environ..

[53] Lebreton L.C.M., van der Zwet J., Damsteeg J.-W., Slat B., Andrady A., Reisser J. (2017). River plastic emissions to the world’s oceans. Nat. Commun..

[54] Rodrigues S.M., Almeida C.M.R., Silva D., Cunha J., Antunes C., Freitas V., Ramos S. (2019). Microplastic contamination in an urban estuary: Abundance and distribution of microplastics and fish larvae in the Douro estuary. Sci. Total Environ..

[55] Holmes L.A., Turner A., Thompson R.C. (2014). Interactions between trace metals and plastic production pellets under estuarine conditions. Mar. Chem..

[56] Rochman C.M., Hentschel B.T., Teh S.J. (2014). Long-term sorption of metals is similar among plastic types: Implications for plastic debris in aquatic environments. PLoS ONE.

[57] Lu K., Quiao R., An H., Zhang Y. (2018). Influence of microplastics on the accumulation and chronic toxic effects of cadmium in zebrafish (*Danio rerio*). Chemosphere.

[58] Wen B., Jin S.R., Chen Z.Z., Gao J.Z., Liu Y.N., Liu J.H., Feng X.S. (2018). Single and combined effects of microplastics and cadmium on the cadmium accumulation, antioxidant defence and innate immunity of the discus fish (*Symphysodon aequifasciatus*). Environ. Pollut..

[59] Salgado J.P., Cabral H.N., Costa M.J. (2004). Feeding ecology of the gobies *Pomatoschistus minutus* (Pallas, 1770) and *Pomatoschistus microps* (Krøyer, 1838) in the upper Tagus estuary, Portugal. Sci. Mar..

[60] Ferreira P., Fonte E., Soares M.E., Carvalho F., Guilhermino L. (2016). Effects of multi-stressors on juveniles of the marine fish *Pomatoschistus microps*: Gold nanoparticles, microplastics and temperature. Aquat. Toxicol..

[61] Monteiro M., Quintaneiro C., Nogueira A.J.A., Morgado F., Soares A.M.V.M., Guilhermino L. (2007). Impact of chemical exposure on the fish *Pomatoschistus micropsis* Krøyer (1838) in estuaries of the Portuguese Northwest coast. Chemosphere.

[62] Vieira L.R., Soares A.M.V.M., Morgado F., Guilhermino L. (2009). Acute effects of copper and mercury on the estuarine fish *Pomatoschistus microps*: Linking biomarkers to behaviour. Chemosphere.

[63] Fonte E., Ferreira P., Guilhermino L. (2016). Temperature rise and microplastics interact with the toxicity of the antibiotic cefalexin to juveniles of the common goby (*Pomatoschistus microps*): Post-exposure predatory behaviour, acetylcholinesterase activity and lipid peroxidation. Aquat. Toxicol..

[64] de Sá L.C., Luís L.G., Guilhermino L. (2015). Effects of microplastics on juveniles of the common goby (*Pomatoschistus microps*): Confusion with prey, reduction of the predatory performance and efficiency, and possible influence of developmental conditions. Environ. Pollut..

[65] Guimarães L., Medina M.H., Guilhermino L. (2012). Health status of *Pomatoschistus microps* populations in relation to pollution and natural stressors: Implications for ecological risk assessment. Biomarkers.

[66] Baeta A., Vieira L.R., Lírio A.V., Canhoto C., Marques J.C., Guilhermino L. (2017). Use of stable isotope ratios of fish larvae as indicators to assess diets and patterns of anthropogenic nitrogen pollution in estuarine ecosystems. Ecol. Indic..

[67] Medina-Gómez I., Kjerfve B., Mariño I., Herrera-Silveira J. (2014). Sources of salinity variation in a coastal lagoon in a Karst landscape. Estuar. Coasts.

[68] OECD (1992). Test No. 203: Fish, Acute Toxicity Test. OECD Guidelines for the Testing of Chemicals, Section 2.

[69] Miranda Maciel T.F. (2014). Toxic Effects of Cadmium, Alone and in Combination with Microplastics, on Early Juveniles of the Common Goby (*Pomatoschistus microps*) in Relation to Previous Long-Term Exposure to Environmental Contamination. Master’s Thesis.

[70] Avio C.G., Gorbi S., Regoli F. (2017). Plastics and microplastics in the oceans: From emerging pollutants to emerged threat. Mar. Environ. Res..

[71] Usero J., Izquierdo C., Morillo J., Gracia I. (2003). Heavy metals in fish (*Solea vulgaris*, *Anguilla anguilla* and *Liza aurata*) from salt marshes on the southern Atlantic coast of Spain. Environ. Int..

[72] Diop M., Howsam M., Diop C., Cazier F., Goossens J.F., Diouf A., Amara R. (2016). Spatial and seasonal variations of trace elements concentrations in liver and muscle of round sardinelle (*Sardinella aurita*) and Senegalese sole (*Solea senegalensis*) along the Senegalese coast. Chemosphere.

[73] de Lima D., Roque G.M., de Almeida E.A. (2013). In vitro and in vivo inhibition of acetycholinesterase and carboxylesterase by metals in zebrafish (*Danio rerio*). Mar. Environ. Res..

[74] Monteiro M., Quintaneiro C., Morgado F., Soares A.M.V.M., Guilhermino L. (2005). Characterization of the cholinesterases presente in head tissues of the estuarine fish *Pomatoschistus micropsis*: Application to biomonitoring. Ecotoxicol. Environ. Saf..

[75] Bradford M. (1976). A rapid and sensitive method for the quantification of microgram quantities of protein utilizing the principle of protein-dye binding. Anal. Biochem..

[76] Guilhermino L., Lopes M.C., Carvalho A.P., Soares A.M.V.M. (1996). Acetylcholinesterase activity in juveniles of *Daphnia magna* Straus. Bull. Environ. Contam. Toxicol..

[77] Ellman G.L., Courtney K.D., Andres V., Feather-Stone R.M. (1961). A new and rapid colorimetric determination of acetylcholinesterase activity. Biochem. Pharmacol..

[78] Habig W.H., Pabst M.J., Jakoby W.B. (1974). Glutathione-S-transferases, the first enzymatic step in mercapturic acid formation. J. Biol. Chem..

[79] Frasco M.F., Guilhermino L. (2002). Effects of dimethoate and betanaphthofavone on selected biomarkers of *Poecilia reticulata*. Fish Physiol. Biochem..

[80] Ohkawa H. (1979). Assay for lipid peroxides in animal tissues by thiobarbituric acid reaction. Anal. Biochem..

[81] Bird R.P., Draper A.H. (1984). Comparative studies on different methods of malondyhaldehyde determination. Methods Enzymol..

[82] Torres M.A., Testa C.P., Gaspari C., Masutti M.B., Panitz C.M.N., Curi-Pedrosa R., de Almeida E.A., Di Mascio P., Wilhelm D. (2002). Oxidative stress in the mussel Mytella gyyanensis from polluted mangroves on Santa Catarina Island. Braz. Mar. Pollut. Bull..

[83] Zar J.H. (1999). Biostatistical Analysis.

[84] Finney D.J. (1971). Probit Analysis.

[85] Gelli F., Cicero A.M., Melotti P., Roncarati A., Pregnolato L., Savorelli F., Palazzi D., Gasazza G. (2004). A proposal of method to evaluate the quality of marine waters: Optimization of 7 days bioassays using *Dicentrarchus labrax* (L.) juveniles. Chem. Ecol..

[86] Thophon S., Kruatrachue M., Upatham E.S., Pokethitiyook P., Sahaphong S., Jaritkhuan S. (2003). Histopatological alterations of white seabass, *Lates calcarifer*, in acute and subchronic cadmium exposure. Environ. Pollut..

[87] Sassi A., Darias M.J., Said K., Messaoudi I., Gisbert E. (2013). Cadmium exposure affects the expression of genes involved in skeletogenesis and stress response in gilthead sea bream larvae. Fish Physiol. Biochem..

[88] Zhang T., Yang M., Pan H., Li S., Ren B., Ren Z., Xing N., Qi L., Ren Q., Xu S. (2017). Does time difference of the acetylcholinesterase (AChE) inhibition in different tissues exist? A case study of zebra fish (*Danio rerio*) exposed to cadmium chloride and deltamethrin. Chemosphere.

[89] Pino J.D., Zeballos G., Anadon M.J., Díaz M.J., Moyano P., Díaz G.G., García J., Lobo M., Frejo M.T. (2016). Muscarinic M1 receptor partially modulates higher sensitivity to cadmium-induced cell death in primary basal forebrain cholinergic neurons: A cholinesterase variants dependent mechanism. Toxicology.

[90] Alsop D., Wood C.M. (2013). Metal and pharmaceutical mixtures: Is ion loss the mechanism underlying acute toxicity and widespread additive toxicity in zebrafish?. Aquat. Toxicol..

[91] Moyano P., de Frias M., Lobo M., Anadon M.J., Sola E., Pelayo A., Díaz M.J., Frejo M.T., Del Pino J. (2018). Cadmium induced ROS alters M1 and M3 receptors, leading to SN56 cholinergic neuronal loss, through AChE variants disruption. Toxicology.

[92] Garcia-Santos S., Fontaínhas-Fernandes A., Wilson J.M. (2006). Cadmium tolerance in the Nile tilapia (*Oreochromis niloticus*) following acute exposure: Assessment of some ionoregulatory parameters. Environ. Toxicol..

[93] Morcillo P., Esteban M.A., Cuesta A. (2016). Heavy metals produce toxicity, oxidative stress and apoptosis in the marine teleost fish SAF-1 cell line. Chemosphere.

[94] Qi L., Ma J., Song J., Li S., Cui X., Peng X., Wang W., Ren Z., Han M., Zhang Y. (2017). The physiological characteristics of zebra fish (*Danio rerio*) based on metabolism and behavior: A new method for the online assessment of cadmium stress. Chemosphere.

[95] Frasco M.-F., Fournier D., Carvalho C., Guilhermino L. (2005). Do metals inhibit acetylcholinesterase (AChE)? Implementation of assay conditions for the use of AChE activity as biomarker of metal toxicity. Biomarkers.

[96] Pretto A., Loro V.L., Morsch V.M., Moraes B.S., Menezes C., Clasen B., Hoehne L., Dressler V. (2010). Acetylcholinesterase activity, lipid peroxidation, and bioaccumulation in silver catfish (*Rhamdia quelen*) exposed to cadmium. Arch. Environ. Contam. Toxicol..

[97] Gupta R., Shukla R.K., Chandravanshi L.P., Srivastava P., Dhuryya Y.K., Shanker J., Singh M.P., Pant A.B., Khanna V.K. (2017). Protective role of quercetin in cadmium-induced cholinergic dysfunctions in rat brain by modulating mitochondrial integrity and MAP kinase signaling. Mol. Neurobiol..

[98] Vivek K.G., Abhishek K., Nikhat J.S., Bechan S. (2016). Rat brain acetyl cholinesterase as a biomarker of cadmium induced neurotoxicity. Open Access J. Toxicol..

[99] Naïja A., Kestemont P., Chénais B., Haouas Z., Blust R., Helal A.N., Marchand J. (2017). Cadmium exposure exerts neurotoxic effects in peacock blennies *Salaria pavo*. Ecotoxicol. Environ. Saf..

[100] Rani S., Gupta R.K., Yadav J. (2017). Heavy metal induced alterations in acetylcholinesterase activity of Indian major carps. J. Entomol. Zool. Stud..

[101] Gill T.S., Tewari H., Pande J. (1991). In vivo and in vitro effects of cadmium on selected enzymes in different organs of the fish *Barbus conchonius* ham. (Rosy Barb). Comp. Biochem. Physiol. Part C.

[102] Souid G., Souayed N., Yaktiti F., Maaroufi K. (2013). Effect of acute cadmium exposure on metal accumulation and oxidative stress biomarkers of *Sparus aurata*. Ecotoxicol. Environ. Saf..

[103] Jebali J., Banni M., Guerbej H. (2006). Effects of malathion and cadmium on acetylcholinesterase activity and metallothionein levels in the fish *Seriola dumerilli*. Fish Physiol. Biochem..

[104] Cattani O., Serra R., Isani G., Raggi G., Cortesi P., Carpene E. (1996). Correlation between metallotionein and energy metabolismo in sea bass, *Dicentrarchus labrax*, exposed to cadmium. Comp. Biochem. Physiol..

[105] Mattsson K., Johnson E.V., Malmendal A., Linse S., Hansson L.A., Cedervall T. (2017). Brain damage and behavioural disorders in fish induced by plastic nanoparticles delivered through the food chain. Sci. Rep..

[106] Ribeiro F., Garcia A.R., Pereira B.P., Fonseca M., Mestre N.C., Fonseca T.G., Ilharco L.M., Bebianno M.J. (2017). Microplastics effects in *Scrobicularia plana*. Mar. Pollut. Bull..

[107] Karami A., Golieskardi A., Ho Y.B., Larat V., Salamatinia B. (2017). Microplastics in eviscerated flesh and excised organs of dried fish. Sci. Rep..

[108] Van Cauwenberghe L., Janssen C.R. (2014). Microplastics in bivalves cultured for human consumption. Environ. Pollut..

[109] Santillo D., Miller K., Johnston P. (2017). Microplastics as contaminants in commercially important seafood species. Integr. Environ. Assess. Manag..

[110] Li J., Green C., Reynolds A., Shi H., Rotchell J.M. (2018). Microplastics in mussels sampled from coastal waters and supermarkets in the United Kingdom. Environ. Pollut..

[111] Peixoto D., Pinheiro C., Amorim J., Oliva-Teles L., Guilhermino L., Vieira M.N. (2019). Microplastic pollution in commercial salt for human consumption: A review. Estuar. Coast. Shelf Sci..

[112] Prata J.C. (2018). Airborne microplastics: Consequences to human health?. Environ. Pollut..

[113] Schwabl P., Liebmann B., Köppel S., Königshofer P., Bucsics T., Trauner M., Reiberger T. (2018). Assessment of microplastics concentrations in human stool—Preliminary results of a prospective study. UEG J..

[114] Besseling E., Wang B., Lürling M., Koelmans A.A. (2014). Nanoplastics affects growth of *S. obliquus* and reproduction of *Daphnia magna*. Environ. Sci. Technol..

[115] Pacheco A., Martins A., Guilhermino L. (2018). Toxicological interactions induced by chronic exposure to gold nanoparticles and microplastics mixtures in *Daphnia magna*. Sci. Total Environ..

[116] Martins A., Guilhermino L. (2018). Transgerational effects and recovery of microplastics exposure in model populations of the freshwater cladoceran *Daphnia magna* Straus. Sci. Total Environ..

[117] Foley C.J., Feiner Z.S., Malinich T.D., Höök T.O. (2018). A meta-analysis of the effects of exposure to microplastics on fish and aquatic invertebrates. Sci. Total Environ..

[118] Schirinzi G.F., Pérez-Pomeda I., Sanchís J., Rossini C., Farré M., Barceló D. (2017). Cytotoxic effects of commonly used nanomaterials and microplastics on cerebral and epitelial human cells. Environ. Res..

[119] Karami A., Romano N., Galloway T., Hamzah H. (2016). Virgin microplastics cause toxicity and modulate the impacts of phenanthrene on biomarker responses in African catfish (*Clarias gariepinus*). Environ. Res..

[120] Sussarellu R., Fabioux C., Guyomarch J., Albentosa M., Huvet A., Soudant P. (2016). Exposure of marine mussels *Mytilus* spp. to polystyrene microplastics: Toxicity and influence on fluoranthene bioaccumulation. Environ. Pollut..

[121] Gramby K., Rainieri S., Rasmussen R.R., Kotterman M.J.J., Sloth J.J., Cederberg T.L., Barranco A., Marques A., Larsen B.K. (2018). The influence of microplastics and halogenated contaminants in feed on toxicokinetics and gene expression in European seabass (*Dicentrarchus labrax*). Environ. Res..

[122] Khan F.R., Syberg K., Shashoua Y., Bury N.R. (2015). Influence of polyethylene microplastic beads on the uptake and localization of silver in zebrafish (*Danio rerio*). Environ. Pollut..

[123] Kim D., Chae Y., An Y.-J. (2017). Mixture toxicity of nickel and microplastics with different functional groups on *Daphnia magna*. Environ. Sci. Technol..

