# Supplementary material for: Neurotoxicity, Behavior, and Lethal Effects of Cadmium, Microplastics, and Their Mixtures on Pomatoschistus microps Juveniles from Two Wild Populations Exposed under Laboratory Conditions―Implications to Environmental and Human Risk Assessment
Source: Int J Environ Res Public Health. 2019 Aug 10;16(16):2857. doi: 10.3390/ijerph16162857 (PMC6720622; doi:10.3390/ijerph16162857)
Supplement: Supplementary file 1 [file ijerph-16-02857-s001.pdf]

International Journal of
Environmental Research and Public Health

Supplementary Material

Neurotoxicity, behaviour and lethal effects of cadmium, microplastics and their mixtures on *Pomatoschistus microps* juveniles from two wild populations - implications to environmental and human risk assessment

Tiago Miranda, Luís R. Vieira and Lúcia Guilhermino

Table S1. Nominal and actual concentrations of microplastics in freshly prepared (0 h) and old test medium (96 h) of different treatments of the bioassays carried out with fish from the estuaries of Minho (M-est) and Lima (L-est) fish. The results are the mean (*n* = 3 per treatment) with corresponding standard deviation. The results of the two-way ANOVA carried with each variable to investigate differences in microplastics concentrations among treatments and between bioassays are also indicated. FLUO 0 h—fluorescence at the beginning of the bioassay. CONC 0 h—concentration of microplastics at the beginning of the bioassay. FLUO 96 h—fluorescence at the end of the bioassay. CONC 96 h—concentration of microplastics at the end of the bioassay. DECAY—percentage of decrease of microplastics concentrations. EEC—estimated exposure concentration during the bioassay.

Treatments	FLUO 0h (F units)	CONC 0h (mg/l)	FLUO 96h (F units)	CONC 96h (mg/l)	DECAY (%)
		M-est fish			
MP	23 ± 1	0.18 ± 0.01	18 ± 1	0.11 ± 0.01	34 ± 4
3 mg/l Cd+MP	21 ± 2	0.15 ± 0.02	18 ± 1	0.11 ± 0.01	31 ± 6
6 mg/l Cd+MP	22 ± 1	0.16 ± 0.01	19 ± 1	0.12 ± 0.02	28 ± 10
13 mg/l Cd+MP	22 ± 2	0.17 ± 0.01	17.9 ± 0.4	0.11 ± 0.01	34 ± 3
25 mg/l Cd+MP	22 ± 1	0.17 ± 0.01	18 ± 1	0.12 ± 0.01	30 ± 8
50 mg/l Cd+MP	22 ± 2	0.17 ± 0.01	18 ± 2	0.11 ± 0.02	31 ± 14
		L-est fish			
MP	24 ± 1	0.18 ± 0.02	18 ± 1	0.11 ± 0.01	35 ± 9
3 mg/l Cd+MP	21 ± 2	0.15 ± 0.02	18 ± 1	0.11 ± 0.01	32 ± 6
6 mg/l Cd+MP	22 ± 2	0.16 ± 0.02	18 ± 2	0.11 ± 0.02	33 ± 14
13 mg/l Cd+MP	22 ± 2	0.17 ± 0.03	19 ± 1	0.12 ± 0.01	29 ± 6
25 mg/l Cd+MP	23 ± 1	0.18 ± 0.02	18 ± 1	0.11 ± 0.02	32 ± 9
50 mg/l Cd+MP	22 ± 2	0.17 ± 0.02	18 ± 2	0.11 ± 0.02	32 ± 14
2-ANOVA				Mean MP conc (mg/l)	EEC (mg/l)
Microplastics Concentrations 0 h	Estuary	$F_{1,24} = 0.195$	$p = 0.663$	0 h<br>0.16 ± 0.02	0.14
	Treatments	$F_{1,24} = 1.595$	$p = 0.200$		
	Interaction	$F_{1,24} = 0.047$	$p = 0.999$		
Microplastics Concentrations 96 h	Estuary	$F_{1,24} < 0.000$	$p = 0.983$	96 h<br>0.11 ± 0.01	
	Treatments	$F_{1,24} = 0.185$	$p = 0.965$		
	Interaction	$F_{1,24} = 0.148$	$p = 0.978$		

Table S2. Mortality recorded in fish from the estuaries of Minho (M-est) and Lima (L-est) Rivers in the bioassays testing the effects of cadmium, microplastics and their mixtures. N1—number of individuals per treatment at the beginning of the bioassay. N2—number of dead fish from the M-est. N3—number of dead fish from the L-est. Mort—percentage of mortality. MP—microplastics (0.14 mg/l). 3 Cd—treatment with 3 mg/l of cadmium. 3 Cd + MP – mixture containing 3 mg/l of cadmium + 0.14 mg/l of MP. 6 Cd – treatment with 6 mg/l of cadmium. 6 Cd + MP – mixture containing 6 mg/l of cadmium + 0.14 mg/l of MP. 13 Cd – treatment with 13 mg/l of cadmium. 13 Cd + MP – mixture containing 13 mg/l of cadmium + 0.14 mg/l of MP.

		M-est		L-est	
**Treatment**	**N1**	**N2**	**Mort (%)**	**N3**	**Mort (%)**
Control	9	0	0	0	0
MP	9	0	0	2	22
3 Cd	9	2	22	3	33
3 Cd + MP	9	2	22	4	44
6 Cd	9	4	44	3	33
6 Cd + MP	9	4	44	4	44
13 Cd	9	5	56	4	44
13 Cd + MP	9	5	56	6	67
25 Cd	9	9	100	7	78
25 Cd + MP	9	9	100	9	100
50 Cd	9	9	100	9	100
50 Cd + MP	9	9	100	9	100

Table S3. Results of three-way ANOVA (3-ANOVA) with interactions (fixed factors: estuary, cadmium concentration and microplastics presence) carried out with *Pomatoschistus microps* juveniles from the estuaries of Minho and Lima rivers. Predatory performance—post-exposure predatory performance. AChE activity—Acetylcholinesterase activity. GST activity—Glutathione *S*-transferases activity. LPO levels—lipid peroxidation levels. Est—original estuary of the fish. Cd—cadmium concentration. MP—presence/absence of microplastics. Est × Cd—interaction between estuary and cadmium concentration. Est × MP—interaction between estuary and microplastics. Cd × MP—interaction between cadmium concentration and microplatics. Cd × MP—interaction between cadmium concentration and microplatics. Est × Cd × MP—interaction among estuary, cadmium and microplastics.

Biomarker	Factor	3-ANOVA
Predatory performance	Est	$F_{1, 80} = 6.024$, $p = 0.016$
	Cd	$F_{3, 80} = 23.926$, $p < 0.001$
	MP	$F_{1, 80} = 5.646$, $p = 0.020$
	Est x Cd	$F_{3, 80} = 0.396$, $p = 0.756$
	Est x MP	$F_{1, 80} = 0.442$, $p = 0.508$
	Cd x MP	$F_{3, 80} = 5.827$, $p = 0.001$
	Est x Cd x MP	$F_{3, 80} = 1.150$, $p = 0.034$
AChE activity	Est	$F_{1, 80} = 20.269$, $p < 0.001$
	Cd	$F_{3, 80} = 7.991$, $p < 0.001$
	MP	$F_{1, 80} = 0.248$, $p = 0.620$
	Est x Cd	$F_{3, 80} = 2.117$, $p = 0.105$
	Est x MP	$F_{1, 80} = 15.238$, $p < 0.001$
	Cd x MP	$F_{3, 80} = 9.473$, $p < 0.001$
	Est x Cd x MP	$F_{3, 80} = 2.965$, $p = 0.037$
GST activity	Est	$F_{1, 80} = 0.075$, $p = 0.785$
	Cd	$F_{3, 80} = 2.047$, $p = 0.114$
	MP	$F_{1, 80} = 0.015$, $p = 0.903$
	Est x Cd	$F_{3, 80} = 0.447$, $p = 0.720$
	Est x MP	$F_{1, 80} = 3.128$, $p = 0.081$
	Cd x MP	$F_{3, 80} = 1.190$, $p = 0.319$
	Est x Cd x MP	$F_{3, 80} = 0.219$, $p = 0.813$
LPO levels	Est	$F_{1, 80} = 0.002$, $p = 0.961$
	Cd	$F_{3, 80} = 1.407$, $p = 0.247$
	MP	$F_{1, 80} = 2.606$, $p = 0.110$
	Est x Cd	$F_{3, 80} = 3.450$, $p = 0.020$
	Est x MP	$F_{1, 80} = 3.071$, $p = 0.084$
	Cd x MP	$F_{3, 80} = 3.125$, $p = 0.030$
	Est x Cd x MP	$F_{3, 80} = 0.415$, $p = 0.743$
